# New (Iso)quinolinyl-pyridine-2,6-dicarboxamide G-Quadruplex Stabilizers. A Structure-Activity Relationship Study

**DOI:** 10.3390/ph14070669

**Published:** 2021-07-13

**Authors:** Enrico Cadoni, Pedro R. Magalhães, Rita M. Emídio, Eduarda Mendes, Jorge Vítor, Josué Carvalho, Carla Cruz, Bruno L. Victor, Alexandra Paulo

**Affiliations:** 1 Faculty of Pharmacy, Research Institute for Medicines (iMed.ULisboa), Universidade de Lisboa, Av. Prof. Gama Pinto, 1649-003 Lisboa, Portugal; en.cadoni@gmail.com (E.C.); ermendes@ff.ulisboa.pt (E.M.); 2 Faculty of Sciences, BioISI-Biosystems & Integrative Sciences Institute, University of Lisboa, Campo Grande, C8 bdg, 1749-016 Lisboa, Portugal; prmagalhaes@fc.ul.pt (P.R.M.); fc53371@alunos.fc.ul.pt (R.M.E.); blvictor@fc.ul.pt (B.L.V.); 3 Department of Pharmaceutical Sciences and Medicines, Faculty of Pharmacy, Universidade de Lisboa, Av. Prof. Gama Pinto, 1649-003 Lisboa, Portugal; 4 Department of Pharmacy, Pharmacology and Health Technologies, Faculty of Pharmacy, Universidade de Lisboa, Av. Prof. Gama Pinto, 1649-003 Lisboa, Portugal; jvitor@ff.ulisboa.pt; 5 CICS-UBI-Centro de Investigação em Ciências da Saúde, Universidade da Beira Interior, Av. Infante D. Henrique, 6200-506 Covilhã, Portugal; josueocarvalho@gmail.com (J.C.); carlacruz@fcsaude.ubi.pt (C.C.)

**Keywords:** quinoline, isoquinoline, G-quadruplex ligands, *k-RAS*, *c-MYC*, telomere, SAR, pyridine-dicarboxamide, molecular dynamics

## Abstract

G-quadruplex (G4)-interactive small molecules have a wide range of potential applications, not only as drugs, but also as sensors of quadruplex structures. The purpose of this work is the synthesis of analogues of the bis-methylquinolinium-pyridine-2,6-dicarboxamide G4 ligand 360A, to identify relevant structure–activity relationships to apply to the design of other G4-interactive small molecules bearing bis-quinoline or bis-isoquinoline moieties. Thermal denaturation experiments revealed that non-methylated derivatives with a relative 1,4 position between the amide linker and the nitrogen of the quinoline ring are moderate G4 stabilizers, with a preference for the hybrid h-Telo G4, a 21-nt sequence present in human telomeres. Insertion of a positive charge upon methylation of quinoline/isoquinoline nitrogen increases compounds’ ability to selectively stabilize G4s compared to duplex DNA, with a preference for parallel structures. Among these, compounds having a relative 1,3-position between the charged methylquinolinium/isoquinolinium nitrogen and the amide linker are the best G4 stabilizers. More interestingly, these ligands showed different capacities to selectively block DNA polymerization in a PCR-stop assay and to induce G4 conformation switches of hybrid h-Telo G4. Molecular dynamic simulations with the parallel G4 formed by a 21-nt sequence present in *k-RAS* gene promoter, showed that the relative spatial orientation of the two methylated quinoline/isoquinoline rings determines the ligands mode and strength of binding to G4s.

## 1. Introduction

With the growing trend in the incidence of cancer [1], the need for developing effective and specific drugs to target cancer cells, for a less toxic and invasive therapy, is becoming increasingly important. One solution under debate is the modulation of gene expression by small molecules, supported by the evidence of the role in oncogenesis played by specific proto-oncogenes. In fact, mutations and translocations occurring within certain proto-oncogenes, such as *c-MYC* [2,3], *k-RAS* [4,5], and *c-KIT* [6], have been frequently reported as a primary cause of cancer onset in several malignancies and for this reason, they have been widely studied as potential therapeutic targets [6,7,8].

It was demonstrated that the promoter regions of these genes, notably rich in guanine residues, are prone to fold into G-Quadruplexes (G4s) [9,10,11,12]. These non-canonical structures arise when four guanines of a G-rich tract are bound together via Hoogsteen hydrogen-bonding, forming the so-called G-quartet or G-tetrad. G4s are formed when two or more tetrads are stacked on top of each other, in an arrangement further stabilized by the presence of a coordinating cation, generally potassium [13]. Besides in proto-oncogenes, G4-forming sequences have also been identified in various other areas of the genome, particularly in the telomeric region [14]. The formation of telomeric G4 could also constitute an obstacle to the action of telomerase, the enzyme which re-synthesizes the terminal part of the chromosomes and has a central role in the aging process and in cancer development [15,16]. The high abundance of G4-forming sequences at the promoter level of genes involved in carcinogenesis, their prevalence in telomeric DNA regions, and their presence in RNA transcripts, suggests an important role of the G4 in the fine regulation of gene expression [17,18,19] as well as post-transcriptional and epigenetic modulation [20]. Therefore, the research efforts focused in small-molecules and oligonucleotide derivatives [21] that induce and stabilize G4-formation, has been seen as a promising approach to identify new therapeutic agents [19,22,23,24,25]. This is of utmost importance, considering the numerous evidences of G4-formation *in vivo*, which suggests a concrete physiological role of these structures in cells [26,27,28].

Among the small-molecule binders, an established family of ligands based on the bis-quinolinyl skeleton, was shown to have a high affinity and selectivity for G4 structures [24]. The use of pyridine or naphthyridine as a central module, combined with a carboxamide with bond rotation impairment, forces the ligand to adopt a highly beneficial V-shape conformation, consequently maximizing the interaction with the target G4 [15,29,30,31]. A paradigmatic model of this chemotype is given by the ligand 360A (**2a**), a *N*-methylbisquinolinium-pyridine-2,6-dicarboxamide, which was shown to be a potent and selective G4-stabilizer. The molecule displayed a binding preference to telomeric G4 (h-Telo), both *in vitro* and in human cells, and antiproliferative effects at a low micromolar concentration in telomerase-positive human cancer cell lines [15,32]. As a possible mechanism of action, Boussin and colleagues proposed that the ligand is able to induce Rad51-dependent aberrations at the telomeric level (telomere loss and doublet), and promote sister telomere fusions upon G4-stabilization [33]. 

Circular dichroism (CD) studies with **2a** showed that the V-shaped structure, which was suggested to be maintained by the formation of intramolecular hydrogen bonds between the pyridine nitrogen and the NH- carboxamide linkers, can vary with the type of cations and anions present in solution [29,34]. Additionally, it was shown that the ligand is able to kick-off a potassium ion from the h-Telo G4. This study provided additional insights regarding the interaction of the ligand with the h-Telo G4. The observed ion ejection may induce either a G4 conformational change to an antiparallel structural arrangement, or the disruption of a quartet of the hybrid G4 [35]. 

Later studies also performed by CD, suggested that the binding of **2a** to h-Telo G4 is accomplished in a 2:1 ligand:DNA stoichiometry [36]. Besides its well-known preferential affinity to h-Telo [32], **2a** has been reported also to bind to other relevant G4-forming sequences, such as those in promoter region of *c-MYC* gene, for which molecular modelling studies suggest a end-stacking mode of binding [37]. Moreover, Van Dyck and co-workers reported its successful use in lymphoblastoid cell lines with the modulation of p53 mRNA transcripts [38]. 

Given the well-established nature of **2a** as G4-ligand, and the simplicity and affordability of its synthesis, a wide range of applications featuring this ligand and its derivatives were reported by numerous research groups active in the field. For example, derivatives of **2a** were successfully employed as alkylating and cross-linking agents of G4-DNA, either by employing visible light for the efficient photo-alkylation of *c-KIT* and *c-MYC* G4 DNA, in the presence of a proximate G4-binding photosensitizer, or for the UV-induced photo-crosslinking to h-Telo when linked to benzophenone or phenyl-azide moieties [39,40]. Other applications featuring **2a** derivatives were extended beyond the canonical therapeutic context for which this ligand was initially designed. For example, this G4 ligand was also used in the development of fluorophore-ligand conjugates applied to fluorescence sensing of G4-DNA [41], in optical probes for G4-sensing, in functionalization with biotin tag to build a bait-molecule for fishing, in the identification of binding-DNA sequences [42], and even as an efficient anion fluorescence sensor [43]. Moreover, dimeric derivatives of **2a**, featuring a higher binding affinity for telomeric G4-DNA, were also reported, increasing the ability of the ligand to target contiguous quadruplex structures [36,44].

Despite the numerous bis-quinoline derivatives reported as G4 ligands (reviewed in [24]), a systematic study elucidating the structure-stabilizing activity relationship (SAR) of the pyridine-2,6-dicarboxamide family of ligands is still missing. In this work, we aimed to shed light on the SAR of various bis-(iso)quinolinyl-pyridine-2,6-dicarboxamides, testing their ability to efficiently stabilize G4-DNA sequences of relevant human genome regions (*k-RAS*, *c-MYC*, h-Telo) over double-stranded DNA sequences. Thermal denaturation experiments of the targeted sequences in the presence of ligands, circular dichroism (CD)-titration experiments, biochemical assays (PCR-stop assay), as well as molecular modelling studies were used to study the influence of the carboxamide linker position in the quinoline/isoquinoline skeleton on the binding of the ligand to different G4 structures.

## 2. Results and Discussion

### 2.1. Synthesis of Pyridine-2,6-dicarboxamide Derivatives

**Scheme 1:**
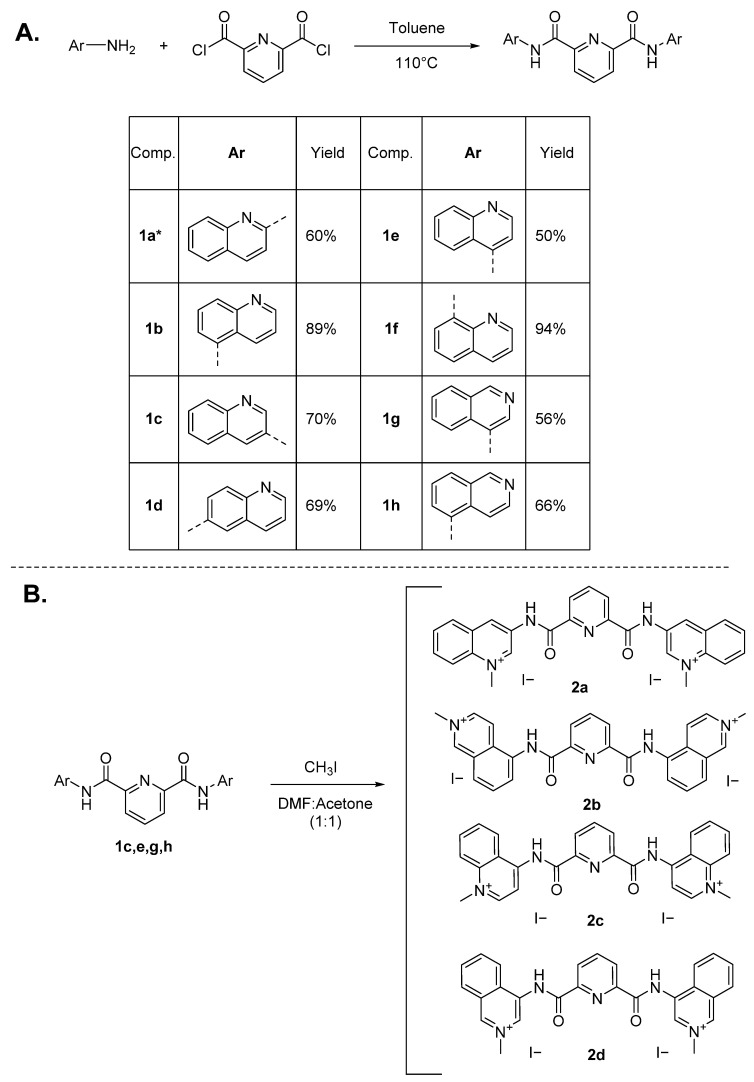
Synthetic route for pyridine-2,6-dicarboxamide derivatives. (**A**). Toluene, 110 °C in reflux, overnight. (**B**). CH_3_I, DMF:acetone (1:1), 1–5 days, RT. * For compound **1a**, another synthetic route was followed, refluxing the mixture in THF as solvent in presence of DIPEA (please refer to material and methods section).

In a first step, a library of simple pyridine-2,6-dicarboxamides (**1b**–**h**) was prepared by reacting 2,6-pyridinedicarbonyl dichloride with the respective aminoquinoline or amino-isoquinoline in toluene, at reflux temperature (Scheme 1A), according to previous literature reports [43,45]. Compound **1a** was prepared by reaction of 2-aminoquinoline with the dichloride using THF (dry) as solvent, in presence of DIPEA as a proton scavenger. The compounds were obtained pure with a yield of 50–94%. Then, *N*-methylation of **1a**–**h** was attempted by reaction with CH_3_I at room temperature in a solvent mixture of acetone:DMF 1:1, followed by filtration and washing of the formed yellow precipitates with ice-cold MeOH. [43] In these conditions, only compounds **1c**, **1e**, **1g** and **1h** afforded, without further purification and with yields of 51–62%, the pure quinolinium iodide salts **2a**, **2c**, **2d**, and **2b**, respectively (Scheme 1B). The synthesis of compounds **2b**–**d** is here reported for the first time.

### 2.2. G4 Thermal Stabilization Induced by Compounds

As a first step to evaluate the interaction of the synthesized molecules to G4-DNA and to have an idea of the stabilization effect of these molecules, a Fluorescence Resonance Energy Transfer (FRET) melting assay was initially performed on TAMRA/FAM-labelled oligonucleotides (Please refer to Supplementary Table S1 for the sequences). Although methylated compounds generally performed better in terms of stabilization, we found that derivatives with amide linker in positions 6 and 4 of quinoline moiety (compounds **1d** and **1e**, respectively) showed a modest stabilizing activity of promoter *k-RAS* G4 (ΔT_m_ = 8.0 °C) and h-Telo (ΔT_m_ = 18.0 and 11.8 °C, respectively) at a concentration of 5 μM (Table 1 and Supplementary Figure S1). It should be noted that these compounds share the same relative 1,4 position between the amide linker and the nitrogen of the quinoline ring, which may contribute to an increased binding to the G4-DNA as a result of the additional basicity of quinoline nitrogen and its consequent extended protonation at pH 7.4.

Upon insertion of a positive charge, all the *N*-methylquinolinium derivatives tested (**2a**–**d**) displayed preferred stabilization of the G4 sequences when compared to duplex DNA (T-loop). Most compounds showed a good G4 stabilization with concentrations starting on 1 μM or 5 eq (Table 2), whereas **2a** showed a good capacity to stabilize G4 at even lower concentrations, as already reported [15]. This result suggests an increased interaction of the ligand with the negatively charged DNA backbone, as reported by Pradeepkumar and co-workers for a naphthyridine-bisquinolinium derivative [30].

To further validate the stabilization induced by **2a**–**d**, CD-melting assays were performed by following the G4 denaturation at the maximum CD wavelength, confirming the results previously obtained in FRET-melting assay (Table 3). FRET and CD melting were employed as complementary techniques to study the ligand-induced thermal stabilization of the ligands. As FRET uses dyes to measure the ΔTm, it is a common trend in this type of studies to use an additional technique to confirm the results, such as UV and CD melting, as occasionally the ΔTm values observed are due to unspecific interaction of the ligands with the dye, rather than direct G4 binding [46]. Overall, a concentration-dependent effect on the stabilization was observed for most ligands as higher concentrations led to a greater thermal stabilization of the G4 structure. In general, methylated compounds display a higher affinity for parallel G4, as shown by the higher ΔT_m_ values obtained in the presence of *c-MYC* and *k-RAS* sequences as compared to h-Telo. For compound **2a** we performed CD melting experiments at the new CD max after structural transition (262 nm) but could not obtain a sigmoidal melting curve, nor a two-state transition. This suggests that the newly obtained conformation is highly stable, probably due to a strong stabilization effect induced by the ligand.

Regarding *c-MYC* parallel G4, all ligands beside **2b** stabilized the structure for more than 20 °C, even at low concentrations, as is the case of **2a**. In the case of *k-RAS* parallel G4, similar results were obtained, with **2b** being the less potent stabilizer, and the remaining ligands presenting ∆T_m_ values greater than 20 °C. *k-RAS* was indeed the most stabilized G4 structure when compared to *c-MYC* and h-Telo, with the latter being the structure for which the ligand-induced thermal stabilization was less evident. 

Overall, both techniques indicate for all G4-DNA structures, an induced thermal stabilization activity trend of **2a** > **2d** > **2c** > **2b**. Also, the relative position of the quinoline nitrogen and the amide -NH, seems to play a role in G4 stabilization. The two most active compounds (**2a** and **2d**) show the common structural feature of having a relative 1,3-position between the charged methyl-quinolinium/isoquinolinium nitrogen and the amide linker.

**Figure 1:**
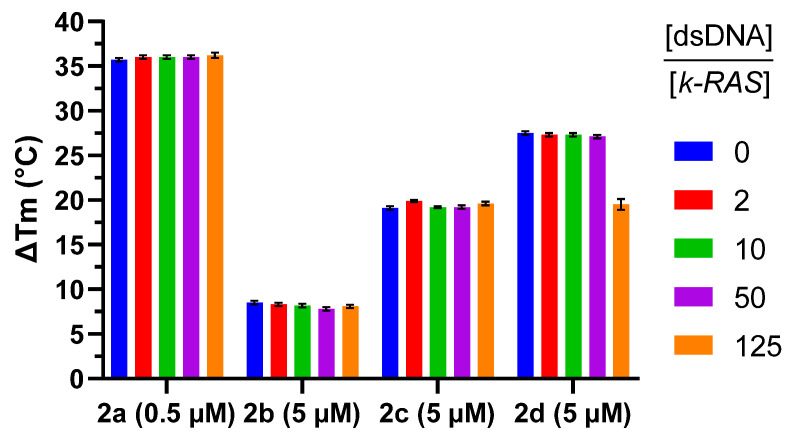
FRET-melting competition assay results for compounds **2a**–**d** at 0.5 μM (**2a**) and 5 μM (**2b**–**d**) complexed with *k-RAS* G4 (0.2 μM), challenged with increasing concentrations of non-labeled 26ds DNA (dsDNA) (0.4 to 25 μM) competitor. Experiments performed in potassium cacodylate buffer (60 mM K^+^, pH 7.4).

Finally, the selectivity of methylated compounds for G4-DNA over double-stranded (ds) DNA, was assessed. For doing so, T_m_ of the FAM/TAMRA-labelled G4 sequence of *k-RAS* complexed with the ligands was recorded in presence of increasing concentrations of competing dsDNA, up to 125-fold. For all the tested compounds, the presence of the competing DNA showed neglectable destabilization of the G4-ligand complex, thus confirming the high selectivity of the compounds for G4-DNA (Figure 1).

### 2.3. G4 Conformational Changes Induced by G4 Ligands ***2a**–**d***

**Figure 2:**
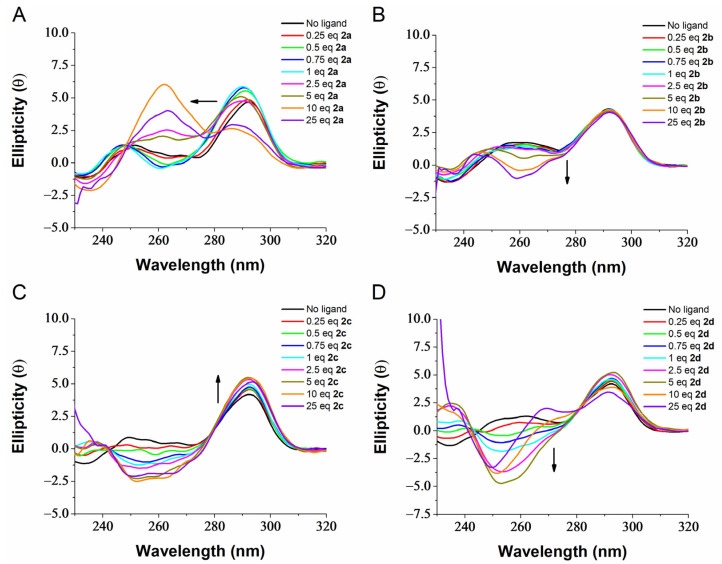
CD titration of h-Telo in presence of increasing equivalents (0–25) of compound **2a** (**A**), **2b** (**B**), **2c** (**C**), **2d** (**D**), at a concentration of 10 µM, performed in 20 mM lithium cacodylate containing 10 mM KCl and 90 mM LiCl.

To retrieve additional information about the binding of the best ligands to G4s, we performed CD-titration experiments. Sequence h-Telo folds into a hybrid-type G4 structure in K^+^ solution as suggested by its characteristic CD signature containing a positive band at 290 nm, a shoulder peak at 265 nm and a weak negative at around 240 nm (Figure 2) [47]. CD-titration experiments on h-Telo suggest that ligand **2a** is able to induce conformational switch upon binding to the target, switching from a hybrid (3+1) topology to a parallel topology (appearance of the typical signature λ_max_ = 264 nm) (Figure 2A). These results contrast with what has been reported previously by Marchand and colleagues [35] where **2a** was suggested to convert h-Telo from a hybrid-type to an antiparallel topology in K^+^ solution. However, the sequence used was longer (24 nt) than that used in our study (21 nt) and the potassium concentration was lower (10 mM K^+^). It is well known that sequence composition, length and salt concentration strongly affect the G4 topology of telomeric G4s [47,48,49]. Surprisingly, this behavior was not preserved by the other methylated derivatives, as even in large ligand excess, the characteristic signature of hybrid G4 (λ_max_ = 290 nm) is maintained. Ligands **2b** and **2c** seem to interact with h-Telo as suggested by the slight ellipticity alteration, particularly around 260 nm, but the overall topology seems to be maintained (Figure 2B,C). In the case of ligand **2d** (Figure 2D), the G4 topology seems to be interconverted into an antiparallel structure as suggested by the strong negative band at 260 nm and a weak positive one around 240 nm following ligand titration. The conformational switches observed may however be incomplete, and mixed conformations can exist in the mix, as suggested by the lack of sharp signals. CD is a technique that lacks high-resolution when compared with others such as NMR or X-ray crystallography, therefore we cannot ascertain whether there are more than one G4 conformation in solution upon ligand binding. 

In the case of *c-MYC*, it folds into a typical parallel G4 as shown by the positive band at 260 nm and the negative band at 240 nm (Supplementary Figure S5). Upon titration with the ligands, only a minor ellipticity variation was observed, suggesting ligand binding without affecting or interconverting the G4 structure. Indeed, *c-MYC* parallel G4 structure is very stable in the K^+^ conditions used and one of the most stable G4s studied [50]. *k-RAS* on its hand, folds into a parallel-stranded G4 structure similarly to *c-MYC* (Figure S6), but upon titration with the ligands, a significant decrease in the ellipticity could be observed, particularly in the case of **2b**, **2c,** and **2d** (Supplementary Figure S6B–D). This signal decrease was accompanied by the appearance of a CD band around 295 nm that suggests structural interconversion and possibly the formation of a hybrid G4 topology.

### 2.4. Taq Polymerase Inhibition by G4-Ligands

**Figure 3:**
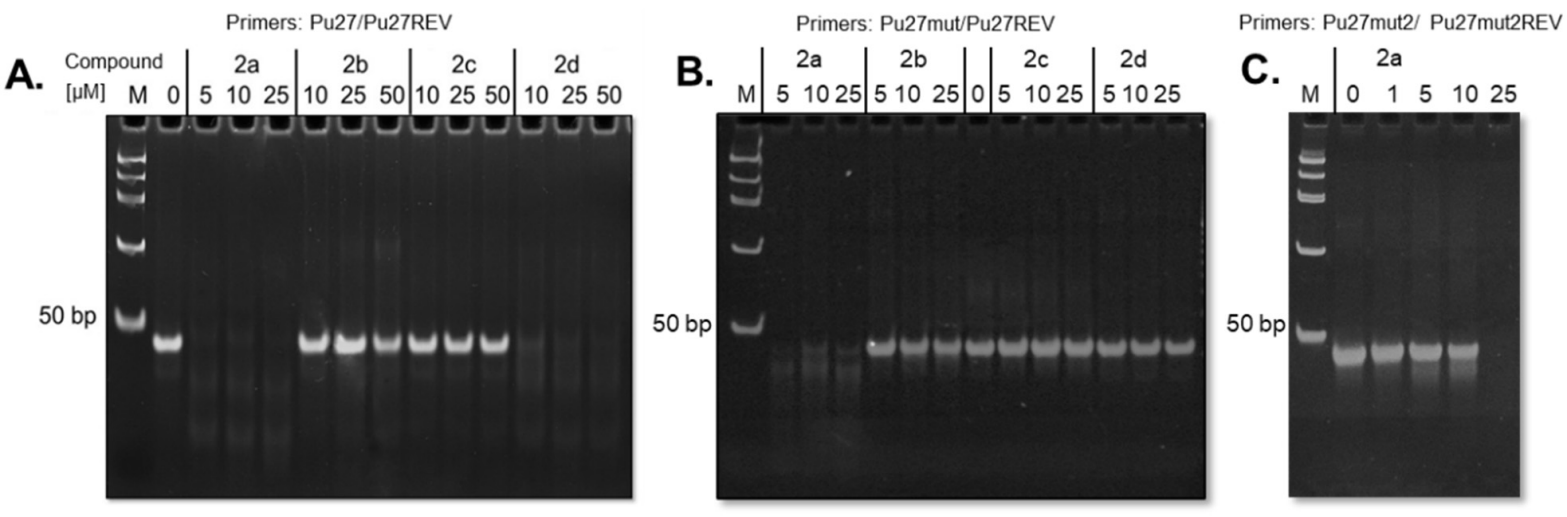
Polyacrylamide gels stained with ethidium bromide showing the effect of compounds on polymerization of DNA constructs. (**A**) Compounds **2a**–**d** in presence of *c-MYC* promoter wild-type sequence (Pu27); (**B**) compounds **2a**–**d** in presence of *c-MYC* promoter sequence mutated in G-tract 3 (Pu27mut); (**C**) increasing concentrations of compound **2a** in presence of *c-MYC* promoter sequence mutated in G-tracts 3 and 5 (Pu27mut2). In absence of compound (0 µM), the 43 bp PCR product is formed. M: PCR molecular weight marker.

To evaluate the ability of the compounds in blocking DNA replication upon G4-stabilization, a polymerase chain reaction (PCR)-stop assay using the G4-forming 27-nucleotide sequence (Pu27) present in the wild-type promoter region of the *c-MYC* oncogene (see sequences in Supplementary Table S3) was performed according to the methodology previously reported [51]. The test is commonly used to evaluate the concentration of ligand necessary to induce the more stable loop-isomer involving G-tracts 2-3-4-5 and block DNA polymerase activity by inhibiting DNA hybridization at G-tract 5 [51]. Consistently with FRET-melting experiments, the non-methylated compounds did not show a significant activity up to 50 μM concentration, even in the case of the best stabilizers of the series, **1d** and **1e** (Supplementary Figure S8A). On the other hand, and further confirming the results obtained with FRET and CD-melting analyses, the two more potent methylated compounds, **2a** and **2d,** proved able to block polymerase activity at a concentration of 5 and 10 µM, respectively (Figure 3A). In contrast, the weaker stabilizing ligands **2b** and **2c**, inducing a low/moderate ∆T_m_ in both FRET and CD melting assays, did not display any activity up to a concentration of 50 µM, in line to what was obtained for the non-methylated **1d**–**1e**. When a mutated sequence at G-tract 3 is used in place of native Pu27 (G13,14 → A mutation), the primer elongation is maintained in the presence of **2d**, even at high excess of ligand (Figure 3B) similarly to that observed for the related compound 307A [52], thus confirming that the polymerase blockade mechanism is ascribed to G4-stabilization rather than to non-specific interactions of the ligand with the DNA primer or with the enzyme. However, the more potent G4-stabilizer, compound **2a**, was also able to stop polymerization of the mutated sequence (Figure 3B and Supplementary Figure S8B). A possible explanation is the ligand **2a** to be also able to induce the formation of the less stable G4 loop isomer involving G-tracts 1-2-4-5 and in this way block hybridization of primers and subsequent replication by polymerase. To investigate this hypothesis, the CD spectra of Pu27mut sequence were recorded in the PCR buffer, in the absence and presence of **2a**, confirming the capacity of **2a** to induce the formation of a DNA structure with a CD profile suggesting a parallel G4, that is, with a negative band around 240 nm and a positive band around 260 nm (Supplementary Figure S7), as previously reported for the G4 isomer 1-2-4-5 [53,54]. To exclude any unspecific mechanism of polymerization inhibition by **2a**, we performed another assay with a sequence mutated in tract-3 and 5, the Pu27mut2 (see sequence in Supplementary Table S3), this one unable to form a G4. Figure 3C shows that polymerization of this sequence is inhibited only at high concentrations of **2a**. This may be due to ligand binding to hybridized primers or even single strand oligonucleotides, blocking the binding of *Taq* polymerase to the primers. In fact, looking to results of the FRET-melting assay in Table 2, ligand **2a** is the one showing highest affinity to bind and stabilize the DNA duplex structure t-Loop.

### 2.5. Molecular Dynamics

To structurally characterize the interaction of **2a**, **2b**, **2c**, and **2d** ligands with the *k-RAS* molecule, we have performed long replicate MD simulations on a 1:1 ratio. In each replicate simulation, the compounds were randomly placed around the *k-RAS* parallel structure (PDB ID: PDB 5i2v). This was done to assure that each simulation was unbiased from any other replicate simulation. The initial 250 ns were not considered for the data analysis of the simulations, since only after this simulation time the complexes reached an equilibrium conformational state (Supplementary Figure S9).

**Figure 4:**
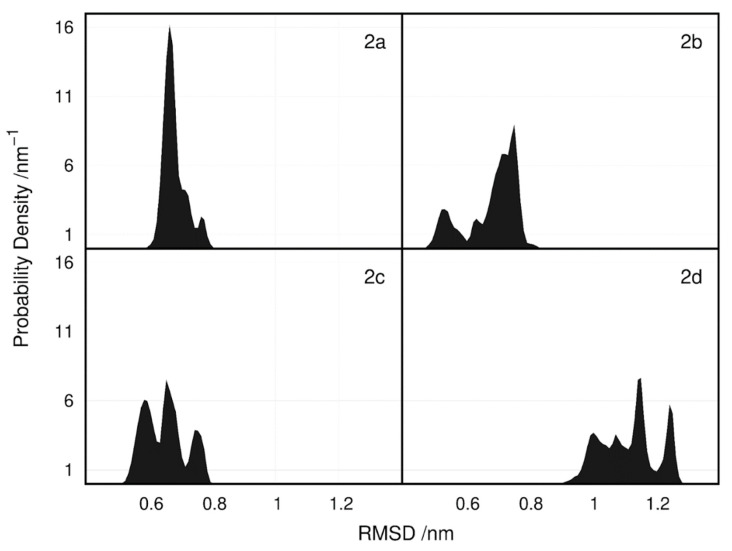
Probability density of the RMSD calculated for all replicate simulations of the different ligands+*k-RAS* complexes. RMSD values were calculated taking as reference, the initial conformation of the complex of the first replicate for each simulated system.

To evaluate the convergence of the conformational space sampled by the ligand: *k-RAS* complex during the MD simulations of each system, we built root mean square deviation (RMSD) histograms calculated from the production trajectories. As can be seen in Figure 4, where we have the probability density of the conformations visited during the MD simulations for each compound, compound **2a** adopts a lower range of conformations, when compared to all the other compounds. While for compound **2a** we can observe one single peak, indicating a highly populated conformation, in compounds **2b**, **2c** and **2d,** at least two highly populated peaks of conformations can be identified. This observation clearly indicates that the convergence of the interaction conformations adopted by compound **2a** is much higher when compared to all the other compounds.

**Figure 5:**
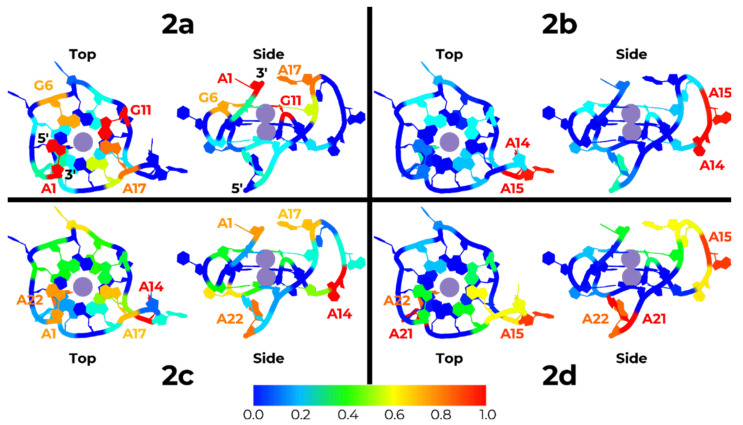
Cartoon representation of the structure of *k-RAS* (top-view on left and side-view on the right) colored according to the normalized number of contacts determined from all the MD simulations (red to dark blue representing the highest number of contacts to the lowest number of contacts).

To identify the preferred interaction configurations between the different compounds and *k-RAS* G4, the number of contacts between the two partners was calculated. Results are presented in Figure 5 and Supplementary Figures S11–S14, where *k-RAS* residues were colored accordingly. Regarding compound **2a**, we can see that both the top and bottom regions of the *k-RAS* molecule, close to its 3′ and 5′ terminal, respectively, are the regions showing the highest number of contacts. By visually inspecting the MD trajectories of the different replicates for this compound, we could verify that in replicates 1, 2, 4, and 5, compound **2a** binds the *k-RAS* at its top, closer to the 3′ terminal, while in replicate 3, the most prevalent configuration shows an interaction of the ligand at the bottom surface, close to the 5′ terminal. Overall, adenine 1, guanine 6 and 11, and adenine 17 showed to be the preferred spots of interaction for this compound. Regarding compound **2b**, we can also clearly identify that the region of *k-RAS* with more interactions was the side loop groove close to adenine 14 and 15. The visual inspection of the trajectories of the different replicate MD simulations indicates that in replicates 2, 3, and 4, compound **2b** interacts preferably with this region of the DNA molecule. This interaction configuration is quite distinct from what was observed in replicate 1 and 5, where the compound **2b** binds respectively the top and bottom of the quadruplex, with the compounds establishing contacts with the guanine tetrad of *k-RAS*. In what concerns compound **2c**, both the top and the bottom regions of k-RAS G4 are the regions with a higher number of contacts. The visual inspection of the different replicate MD simulations indicates that in replicates 1 and 2, compound **2c** binds to the bottom of the G4, while in replicates 4 and 5, the ligand preferably binds at its top. In replicate 3, we could observe that compound **2c** binds also at the side region of the G4 close to residues 12 and 17. Finally, regarding compound **2d**, we clearly see that the preferred region of interaction with *k-RAS* G4 is the bottom region close to the 5′ terminal. The visual inspection of the different trajectories indicates that in replicates 3 and 5 compound **2d** preferably binds the side region of *k-RAS* (between residues 15 and 19), in replicates 2 and 4 the interaction is mostly done at the bottom region close to the 5′ terminal, while in replicate 1 the preference for interactions is observed at the top of *k-RAS* G4, close to the 3′ terminal. Overall, these results confirm the conclusions previously taken from the analysis of the RMSD histograms, where compound **2a** shows a higher convergence of pose interactions to *k-RAS* when compared to all the other tested compounds. 

**Figure 6:**
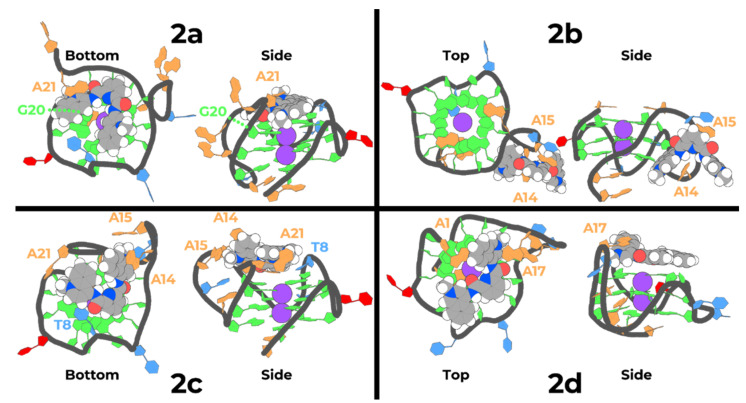
Snapshots of the representative lowest binding energy pose of each compound to parallel *k-RAS* G4 (top or bottom views on the left and side views on the right). The backbone of *k-RAS* G4 molecule is represented as black cartoon, and all the side-chains of the pairs of bases are differently colored with filled lines and sticks: guanines are colored in green, adenines in orange, thymines in blue and cytosines in red.

From the previous analysis, it is however difficult to identify a specific binding pose that could be seen as a representative of interaction between each one of the compounds and *k-RAS* G4. Therefore, we have analyzed the binding free energy of each compound throughout all the replicate simulations to identify a representative lowest binding affinity pose. To achieve this goal, we performed MM/PBSA calculations every 5 ns of simulation for all replicates and systems and identified the part of the trajectory of the replicate where a lower value for the interaction of the compound and the *k-RAS* G4 was observed. Ultimately, from these sets of conformations, we selected a representative of the interaction between *k-RAS* and each compound (Figure 6 and Supplementary Figure S10).

In the selected lowest binding energy representative conformation of compound **2a** with *k-RAS* G4 we can see that the compound stacks in a planar configuration at the bottom region of the G4, close to the 5′ terminal. A perfect stack between the pyridine, the two quinoline groups, and the guanine-rich core of the parallel G4 is achieved. Interestingly, both the positively charged *N*-methylated groups in the two quinolines are facing the outward region of the core of the G4, directed to the negatively charged phosphate groups, evidencing a strong electrostatic attractive effect (see Table 4). Additionally, it is also possible to observe that one of the quinolines is double stabilized by the aromatic rings of guanine 20 and adenine 21, in an intercalating aromatic configuration that restrains the movements of this group, and consequently stabilizes the entire molecule on this conformation.

The previously described interaction observed in compound **2a** is completely distinct from the interaction observed in the representative lowest binding affinity conformation of compound **2b** with *k-RAS*. As can be seen in Figure 6, compound **2b** directly interacts on a side groove of *k-RAS* between adenine 15 and guanine 19. In this conformation, the pyridine group of the compound stacks with adenine 15, with both the isoquinolines showing a 90° rotation of the aromatic groups in respect to the plane of the pyridine group. This configuration allows for one of the isoquinolines to stack with the aromatic region of adenine 14 and with the *N*-methylated group facing the phosphorus of guanine 20. The other isoquinoline faces the solvent, exposing the *N*-methylated group to the environment water molecules. This configuration, although showing a good binding free energy to *k-RAS* (see Table 4), is in a completely different interaction configuration when compared to compound **2a**. Moreover, it is also possible to find interaction configurations of compound **2b** in a similar configuration to the one previously described for compound **2a** (stacked at the top and bottom regions of *k-RAS*). However, these configurations are of much higher binding free energy, which could be correlated with its lower ability to stabilize *k-RAS*. 

Regarding the representative lowest binding affinity configuration for compound **2c** (Figure 6) we can see that, similarly to compound **2a**, this compound stacks at the bottom of the *k-RAS* G4. Both the pyridine and quinoline groups of this compound are stacked with the central guanine tetrads. Additionally, the positive *N*-methylated groups in quinolines are also facing the phosphate backbone of G4, establishing strong favorable electrostatic interactions (Table 4). However, while compound **2a** shows a full planar interaction configuration at top of the G4 molecule, in compound **2c** only one quinoline is fully stacked with adenine 21, while the other quinoline evidences a twisted conformation which ultimately results in a less stable configuration when compared to compound **2a**.

Finally, in what concerns compound **2d**, similarly to what was observed for compounds **2a** and **2c**, the lowest binding affinity representative conformation is found stacking on the top of the *k-RAS* G4 in a very similar configuration as the one adopted by compound **2c**. The major difference observed from these two compounds comes from the more planar conformation with the guanine-rich core of *k-RAS*, which is further stabilized by stacking with adenine 17. Additionally, both the *N*-methylated groups seem to adjust in order to assure that a favorable electrostatic interaction with the negatively charged phosphate groups of the DNA backbone is achieved. This interaction configuration seems to allow compound **2d** to interact and stabilize *k-RAS* to a greater extent when compared to compound **2c.**

To try to correlate the binding free energies of the representative configurations of the different compounds with the experimental melting temperature assay results, we have calculated the average binding free energy of the different compounds with the 50 more similar configurations to the previously analyzed representative solutions (based on RMSD differences). As can be seen in Table 4, compound **2b** stands out as the one showing a higher binding free energy to the *k-RAS* G4. However, one should mention that the interaction configurations showing these binding free energy values correspond to quite different configurations in respect to those observed for all the other compounds. 

Another fact that is possible to identify from the analysis of Table 4, is that the electrostatic energies are the forces that most contribute to the overall binding free energies, which most probably come from the previously described electrostatic interactions observed between the *N*-methylated groups of the quinolines/isoquinolines and the backbone phosphates of the DNA. Overall, the representative interaction configurations identified from the MD simulations for compounds **2a**, **2c** and **2d** show a clear stacking of the pyridine and quinoline/isoquinoline groups with the guanine tetrad at the core of *k-RAS* G4, with the positive *N*-methylated groups of the quinolines/isoquinolines interacting with the negative G4-DNA phosphates. The small conformational differences observed between the different compounds with G4, which consequently are responsible for the determined differences in the binding free energies, seem to come from the degree of achieved planarity between the aromatic groups. Except for compound **2b**, the binding results obtained can directly be correlated with the results from the melting temperature assays previously described, reporting compound **2a** as the one with a higher ability to stabilize *k-RAS* G4 when compared to compounds **2d** and **2c**, respectively. However, one should recall that binding free energies only evaluate the binding and should not be directly correlated with the stabilization effect of the different compounds on the full G4. Therefore, despite the fact that we were able to get a good correlation between the binding affinities and the melting temperature assays for compounds **2a**, **2c** and **2d**, the results obtained for compound **2b** evidence the need for a careful and comparative analysis of the determined binding affinity results and the overall stabilization of the G4 determined by the temperature melting assays.

## 3. Materials and Methods

### 3.1. Synthesis of Compounds

#### 3.1.1. General Procedure A for Synthesis of Bis-quinolinyl/isoquinolinyl-pyridine-2,6-dicarboxamides (**1b**–**h**)

In a round-bottom flask, a mixture of aminoquinoline/amino-isoquinoline (70 mg, 0.60 mmol) and pyridine-2,6-dicarbonyl-dichloride (50 mg, 0.245 mmol) was refluxed overnight in 2.0 mL of freshly dried toluene. The resulting precipitate was recovered by suction filtration, then washed with acetone and a 5% aqueous solution of NaHCO_3_ to obtain the titled compound (**1b–1h**) as a white powder with a yield of 50–95%.

***N*^2^,*N*^6^-di(Quinolin-2-yl)pyridine-2,6-dicarboxamide** (**1a**). 2-aminoquinoline (77.75 mg, 0.539 mmol) was dissolved in 3 mL of THF and 93.92 µL of DIPEA (0.539 mmol) added to the mixture. Pyridine-2,6-dicarbonyl-dichloride (50 mg, 0.245 mmol) was finally added to the solution refluxed for 24H. The solvent was then removed in vacuum and the product purified by flash chromatography (100 Hexane to 3:7 Hexane/EtOAc) to give a light brown powder in a yield of 60%. ^1^H NMR (CDCl_3_) δ 11.27 (s, 2H), 8.63 (d, J = 8.7 Hz, 2H), 8.45 (d, J = 7.8 Hz, 2H), 8.18 (d, J = 9.0 Hz, 2H), 8.08 (t, J = 7.8 Hz, 1H), 7.90 (d, J = 8.4 Hz, 2H), 7.74 (d, J = 7.9 Hz, 2H), 7.64 (t, J = 7.7 Hz, 2H), 7.42 (t, J = 7.5 Hz, 2H). ^13^C NMR (CDCl_3_) δ 162.18, 150.87, 148.54, 146.26, 139.50, 139.00, 130.20, 127.60, 127.44, 126.38, 125.96, 125.51, 114.50. ESI-MS *m*/*z* (100%) 420.2 ([M + H]^+^), HPLC-MS: RT = 3.41 min. Calculated purity 87%. 

***N*^2^,*N*^6^-di(Quinolin-5-yl)pyridine-2,6-dicarboxamide** (**1b**). The title compound was synthesized following the general synthetic procedure A, reacting pyridine-2,6-dicarbonyl-dichloride with 5-aminoquinoline, with a final yield of 89%. ^1^H NMR (DMSO-d6) δ 11.48 (s, 2H, NH), 8.97 (d, J = 2.4 Hz, 2H), 8.57–8.43 (m, 4H), 8.37 (dd, J = 7.2, 7.8 Hz, 1H), 8.02 (d, J = 8.1 Hz, 2H), 7.92–7.77 (m, 4H), 7.64 (dd, J = 9.0, 6.0 Hz, 2H). ^13^C NMR (DMSO-d6) δ 163.21, 151.32, 148.96, 148.59, 140.69, 133.76, 132.75, 129.59, 128.19, 125.96, 125.00, 124.96, 121.92. ESI-MS *m*/*z* (100%) 171.1, 420.2 ([M + H]^+^). HPLC-MS RT = 3.50 min. Calculated purity 98%.

***N*^2^,*N*^6^-di(Quinolin-3-yl)pyridine-2,6-dicarboxamide (1c**). The title compound was synthesized following the general synthetic procedure A, reacting pyridine-2,6-dicarbonyl-dichloride with 3-aminoquinoline, with a final yield of 70%. ^1^H NMR (DMSO-d6) δ 9.39 (d, J = 2.5 Hz, 2H), 8.98 (d, J = 2.5 Hz, 2H), 8.47 (d, J = 7.6 Hz, 2H), 8.36 (dd, J = 7.8, 6.9 Hz, 1H), 8.03 (d, J = 8.1 Hz, 4H), 7.71 (td, J = 9.0, 1,2 Hz, 2H), 7.63 (td, J = 9.0, 1.0 Hz, 2H). ^13^C NMR (DMSO-d6) δ 162.94, 148.93, 146.55, 145.12, 140.72, 132.41, 129.11, 128.87, 128.40, 128.16, 127.64, 126.12, 124.88. ESI-MS *m*/*z* (100%) 171.1, 420.2 ([M + H]^+^). HPLC-MS, RT= 3.19 min. Calculated purity 96%.

***N*^2^,*N*^6^-di(Quinolin-6-yl)pyridine-2,6-dicarboxamide** (**1d**). The title compound was synthesized following the general synthetic procedure A, reacting pyridine-2,6-dicarbonyl-dichloride with 6-aminoquinoline, with a final yield of 69%. ^1^H NMR (DMSO-d6) δ 11.39 (s, 2H, NH), 8.86 (dd, J = 4.2, 1.6 Hz, 2H), 8.73 (d, J = 2.2 Hz, 2H), 8.49 (d, 2H), 8.43 (dd, J = 7.6, 2.3 Hz, 2H), 8.37 (t, J = 8.6, 6.8 Hz, 1H), 8.26 (dd, J = 9.1, 2.3 Hz, 2H), 8.13 (d, J = 9.1 Hz, 2H), 7.56 (dd, J = 8.3, 4.2 Hz, 2H). ^13^C NMR (DMSO-d6) δ 162.53, 150.03, 149.15, 145.48, 140.68, 136.49, 136.40, 129.87, 128.69, 126.12, 125.40, 122.44, 117.72. ESIMS *m*/*z* (100%) 171.1, 420.2 ([M + H]^+^). HPLC-MS, RT = 3.34 min. Calculated purity 96%.

***N*^2^,*N*^6^-di(Quinolin-4-yl)pyridine-2,6-dicarboxamide** (**1e**). The title compound was synthesized following the general synthetic procedure A, reacting pyridine-2,6-dicarbonyl-dichloride with 4-aminoquinoline with a final yield of 50%. ^1^H NMR (DMSO-d6) δ 11.59 (s, 2H, NH), 8.98 (d, J = 4.9 Hz, 2H), 8.53 (d, J = 7.5 Hz, 2H), 8.46- 8.35 (m, 3H), 8.10 (d, J = 8.5 Hz, 2H), 8.01 (d, J = 4.9 Hz, 2H), 7.86 (td, J = 7.8, 1.4 Hz, 2H), 7.75 (td, J = 7.7, 1.4 Hz, 2H). ^13^C NMR (DMSO-d6) δ 163.92, 152.21, 150.16, 149.71, 142.34, 141.83, 131.31, 130.73, 127.82, 127.53, 124.70, 124.21, 117.23. ESI-MS *m*/*z* (100%) 171.1, 420.2 ([M + H]^+^), HPLC-MS, RT = 2.94 min. Calculated purity 98%.

***N*^2^,*N*^6^-di(Quinolin-8-yl)pyridine-2,6-dicarboxamide** (**1f**). The title compound was synthesized following the general synthetic procedure A, reacting pyridine-2,6-dicarbonyl-dichloride with 8-aminoquinoline with a final yield of 94%. ^1^H NMR (CDCl_3_) δ 12.38 (s, 2H, NH), 9.04 (dd, J = 7.0, 2.0 Hz, 2H), 8.60 (d, J = 7.8 Hz, 2H), 8.28 (dd, J = 4.2, 1.7 Hz, 2H), 8.25–8.17 (m, 3H), 7.75–7.59 (m, 4H), 7.35 (dd, J = 8.2, 4.2 Hz, 2H). ^13^C NMR (CDCl_3_) δ 162.01, 149.86, 148.70, 139.60, 139.34, 136.14, 134.42, 128.06, 127.37, 125.43, 122.35, 121.42, 117.28. ESI-MS *m*/*z* (100%) 171.1, 420.2 ([M + H]^+^), HPLC-MS, RT = 3.24 min. Calculated purity 94%

***N*^2^,*N*^6^-di(Isoquinolin-4-yl)pyridine-2,6-dicarboxamide** (**1g**). The title compound was synthesized following the general synthetic procedure A, reacting pyridine-2,6-dicarbonyl-dichloride with 4-amino-isoquinoline with a final yield of 56%. ^1^H NMR (DMSO-d6) δ 11.46 (s, 2H, NH), 9.34 (s, 2H), 8.71 (s, 2H), 8.48 (d, J = 8.4 Hz, 2H), 8.38 (dd, J = 9.0, 6.5 Hz, 2H), 8.25 (d, J = 8.4 Hz, 2H), 8.11 (d, J = 8.4 Hz, 2H), 7.91 (td, J = 8.4, 1.3 Hz, 2H), 7.78 (td, J = 8.1, 1.2 Hz, 2H). ^13^C NMR (DMSO-d6) δ 163.45, 151.47, 148.85, 141.43, 140.73, 140.40, 132.45, 131.32, 128.98, 128.63, 128.35, 126.05, 123.08. ESI-MS *m*/*z* (100%) 171.1, 420.2 ([M + H]^+^), HPLC-MS, RT= 2.89 min. Calculated purity 83%.

***N*^2^,*N*^6^-di(Isoquinolin-5-yl)pyridine-2,6-dicarboxamide** (**1h**). The title compound was synthesized following the general synthetic procedure A, reacting pyridine-2,6-dicarbonyl-dichloride with 5-aminoisoquinoline with a final yield of 66%. ^1^H NMR (DMSO-d6) δ 11.46 (s, 2H, NH), 9.40 (d, J = 1.2 Hz, 2H), 8.60 (d, J = 6.0 Hz, 2H), 8.47 (d, J = 7.7 Hz, 2H), 8.38 (dd, J = 8.8, 6.5 Hz, 1H), 8.18 (d, J = 6 Hz, 2H), 7.99 (dd, J = 8.2, 1.2 Hz, 2H), 7.95 (d, J = 6.0 Hz, 2H), 7.80 (t, J = 8.2 Hz, 2H). 13C NMR (DMSO-d6) δ 163.18, 153.09, 148.93, 143.52, 140.72, 132.74, 132.15, 129.25, 128.77, 127.77, 126.85, 126.00, 116.89. ESI-MS *m*/*z* (100%) 420.2 ([M + H]^+^). HPLC-MS, RT = 3.36 min. Calculated purity 95%.

#### 3.1.2. General Procedure B for Synthesis of Bis-methyl-quinolinium/isoquinolinium-pyridine 2,6-Dicarboxamides (**2a**–**d**)

In a round-bottom flask, 50 mg of pyridine-2,6-dicarboxamide (**1c**, **1e**, **1g**, or **1h**, 0.119 mmol) were dissolved in 1.5 mL of a mixture DMF:Acetone (1:1). 148.5 µL of CH_3_I were added to the reaction mixture, which was stirred for 5 days at room temperature. The resulting yellow precipitate was filtered and washed with cold MeOH to obtain the methylated bis-quinolinium/isoquinolinium iodide (**2a**–**2d**) with a yield of 50–60%.

**3,3′-((Pyridine-2,6-dicarbonyl)bis(azanediyl))bis(1-methylquinolin-1-ium) Iodide** (**2a**) This compound was synthesized from **1c** (0.119 mmol) according to general procedure B, to give the title compound in 61% yield. ^1^H NMR (DMSO-d6) δ 11.83 (s, 2H, NH), 10.13 (s, 2H), 9.67 (s, 2H), 8.62–8.53 (m, 6H), 8.49 (t, J = 7.7 Hz, 1H), 8.24 (t, J = 7.5 Hz, 2H), 8.08 (t, J = 7.6 Hz, 2H), 4.80 (s, 6H). ^13^C NMR (DMSO-d6) δ 162.5, 147.5, 144.7, 141.2, 135.9, 134.5, 134.1, 132.3, 130.5, 129.9, 129.3, 126.5, 122.8, 119.3, 118.5, 49.2. NMR data in agreement with previously reported in [45].). ESI-MS *m*/*z* (100%) 185.2, 448.3 ([M-H-2I]^+^). HPLC-MS, RT = 2.83 min. Calculated purity >90%

**5,5′-((Pyridine-2,6-dicarbonyl)bis(azanediyl))bis(2-methylisoquinolin-2-ium) Iodide** (**2b**) was obtained from 1h (0.119 mmol) according to general procedure B, with a final yield of 56%. ^1^H NMR (DMSO-d6) δ 11.65 (s, 2H, NH), 10.10 (s, 2H), 8.74 (d, J = 6.6 Hz, 2H), 8.65 (d, J = 6.9 Hz, 2H), 8.55–8.38 (m, 7H), 8.18 (t, J = 7.9 Hz, 2H), 4.52 (s, 6H). ^13^C NMR (DMSO-d6) δ 163.36, 151.61, 148.55, 141,11 (Detected via HMQC), 136.19, 134.22, 133.77, 133.34, 131.77, 129.33, 128.29, 126.40, 122.82, 48.46. ESI-MS *m*/*z* (100%) 185.2, 448.3 ([M-H-2I]^+^), HPLC-MS, RT = 2.83 min. Calculated purity 96%.

**4,4′-((Pyridine-2,6-dicarbonyl)bis(azanediyl))bis(1-methylquinolin-1-ium) Iodide** (**2c**) was obtained from **1e** (0.119 mmol) according to general procedure B, with a final 51% yield. ^1^H NMR (DMSO-d6) δ 12.10 (s, 2H), 9.45 (d, J = 6.9 Hz, 2H), 9.11 (d, J = 8.5 Hz, 2H), 8.79 (d, J = 6.8 Hz, 2H), 8.68 (d, J = 7.7 Hz, 2H), 8.61–8.47 (m, 3H), 8.34 (t, J = 7.5 Hz, 2H), 8.08 (t, J = 7.7 Hz, 2H), 4.56 (s, 6H). ^13^C NMR (DMSO-d6) 163.84 (C=O), 150.73 (CH), 148.13 (C), 141.35 (CH), 139.95 (C), 135.76 (CH), 129.09 (CH), 128.19 (CH), 125.91 (CH), 125.60 (C), 121.96 (C), 119.98 (CH), 112.56 (CH), 45.16 (CH3). ESI-MS *m*/*z* (100%) 185.2, 448.3 ([M-H-2I]^+^), HPLC-MS, RT = 2.83 min. Calculated purity 95%.

**4,4′-((Pyridine-2,6-dicarbonyl)bis(azanediyl))bis(2-methylisoquinolin-2-ium) Iodide** (**2d**) was obtained from **1g** (0.119 mmol) according to general procedure B, stirring for 2 days, with a final 51% yield. 

^1^H NMR (DMSO-d6) δ 11.78 (s, 2H), 9.97 (s, 2H), 9.21 (s, 2H), 8.65 (d, J = 8.5 Hz, 2H), 8.58 (d, J = 7.6 Hz, 4H), 8.52–8.46 (m, 1H), 8.39 (t, J = 7.8 Hz, 2H), 8.17 (t, J = 7.7 Hz, 2H), 4.56 (s, 6H). ^13^C NMR (DMSO-d6) δ 163.34, 148.94, 148.17, 141.30 (Detected in HMQC), 136.99, 132.87, 132.84, 132.68, 132.09, 131.10, 128.04, 127.06, 123.94, 48.95. ESI-MS *m*/*z* (100%) 185.2, 448.3 ([M-H-2I]^+^), HPLC-MS, RT = 2.83 min. Calculated purity 97%.

### 3.2. PCR-Stop Assay

The protocol for PCR-Stop assay and the primer design was adapted from a previously reported protocol [52]. For the complete sequence list, refer to Supplementary Table S3. Briefly, for each reaction, 0.5 μL of Taq DNA Polymerase (New England Biolabs (NEB), Inc., Ipswich, MA, USA), 2.0 μL of ThermoPol^®^ buffer (20 mM Tris-HCl, 10 mM (NH4)_2_SO_4_, 10 mM KCl, 2 mM MgSO_4_, 0.1% Triton^®^ X-100, pH 8.8@25 °C, NEB), 1.0 μL of dNTP mix (10 mM each dNTP, NEB), 1.0 μL of each primer (straight + reverse, 25 μM, STAB VIDA, Caparica, Portugal) solutions were added, for a total volume of 5.5 μL. Water, Sigma-HPLC grade, and the tested compound are added to a final volume of 20 μL. PCR conditions: 94 °C for 3 min, followed by 10 cycles of 94 °C 30 s, 58 °C 30 s, and 72 °C 30 s. After the amplification, 3 μL of Gel Loading Dye Bleu (6×, NEB) were added. The amplified products were separated by electrophoresis in a 12% polyacrylamide gel (Acrylamide/Bis 19:1, 40%, NOVEX), TBE 1×. The gel was stained with ethidium bromide and photographed under UV light. PCR Marker (50–766 bp, NEB) was used as molecular weight marker.

### 3.3. FRET-Melting Analysis and Competition Experiments

Working solutions of 5′-FAM/3′TAMRA- labelled oligonucleotides (please refer to Supplementary Table S1 for the complete sequence list) were freshly prepared, diluted with FRET buffer (60 mM potassium cacodylate, pH 7.4) at a final concentration of 20 µM. These were diluted at 0.4 µM, annealed at 95 °C for 10 min in a heating block, then slowly cooled to room temperature (over 1.5 h). Then, 50 µL of annealed DNA solution were mixed with 50 µL of tested compound at the appropriate concentration, in 96-Well RT-PCR plates, with a final DNA concentration of 0.2 µM. Fluorescence readings were taken at intervals of 0.5 °C, in the range 31–95 °C. The advanced curve-fitting function in GraphPad Prism (nonlinear regression fit) was used for the calculation of ΔTm values. Only results with fitting r^2^ values > 0.75 were considered.

For the competition experiments, the melting of the *k-RAS* G4 (0.2 µM) complexed with ligand (5 µM or 0.5 µM) was monitored under the same conditions, including 0.4, 2.0, 10, and 25 µM of non-fluorescent double-stranded competitor 26ds DNA, using the same FRET assay conditions.

### 3.4. CD Titration and Melting Analyses

All CD measurements were performed at 20 °C at 10 µM strand concentration of oligonucleotide in 20 mM lithium cacodylate containing 10 mM KCl and 90 mM LiCl for h-Telo, 20 mM lithium cacodylate containing 100 mM LiCl for *c-MYC*, and 20 mM lithium cacodylate containing 50 mM KCl and 50 mM LiCl for *k-RAS*. The ionic strength was tuned so that the G4 structures would melt around 50 °C. This offers a wider temperature window where ligand-induced thermal stabilization can be studied and enables a more accurate data analysis as good baselines are obtained at both ends (low and high temperature). The sequences were annealed by heating to 95 °C for 10 min followed by cooling on ice for 1 h. For CD titration experiments, a 10 mM stock solution of each ligand was prepared in DMSO and the required volume for each point was added directly to a 1 mm quartz cell containing the oligonucleotide solution. CD spectra were recorded using a Jasco J-815 spectropolarimeter equipped with a Peltier-type temperature control system (model CDF-426S/15), using an instrument scanning speed of 200 nm/min with a response time of 1 s in wavelengths ranging from 200 to 340 nm. The recording bandwidth was 1 nm with 1 nm step size. The spectra were signal averaged over four scans and baseline corrected by subtracting a buffer spectrum.

CD melting spectra were acquired in the temperature range of 20–100 °C, with a heating rate of 1 °C/min by monitoring the ellipticity at 290 and 265 nm for telomeric DNA and oncogene promoters, respectively. Spectra were acquired in the presence of 1, 5, 10 and 25 molar equivalents of ligand. CD melting spectra were acquired in 20 mM lithium cacodylate buffer supplemented with KCl and LiCl as described above, keeping a constant ionic strength of 100 mM (total salt concentration). Data were converted into fraction folded plots, fit to a Boltzmann distribution and the melting temperatures determined from the two-state transition model (OriginPro 8).

### 3.5. Molecular Modeling

#### 3.5.1. Compound Structure Preparation

The 3D structures of **2a**, **2b**, **2c**, and **2d** compounds were initially generated using the MarvinSketch (version 20.13) software from Chemaxon (http:www.chemaxon.com, accessed on 1 July 2020). The resulting geometries were then optimized at the B3LYP/6-31G(d) level of theory [55,56,57,58,59], using Gaussian 09, Rev A.2 [60]. To derive the partial atomic charges for each compound, we have used the strategy described for GAFF [57,61], with the molecular electrostatic potentials (ESP) created for all elements at the HF/6-31G(d) [62,63,64] level of theory. The overall charge of each compound was set to +2. The atomic partial charges were afterwards derived following the restrained ESP (RESP) [65] procedure, using the antechamber [66] module implemented in AmberTools19 [67].

#### 3.5.2. Molecular Dynamics Simulations

All molecular mechanics/dynamics (MM/MD) simulations were performed using the GROMACS 2018.6 software package [68,69,70] as well as the OL15 refinement of the Amber14sb force field [71,72,73,74].

The initial structure of the *k-RAS* molecule was taken from PDB 5i2v [75]. Four different systems were built, each consisting of the quadruplex and one of the four compounds previously parameterized. Amber topologies were built for each compound using the tleap module in AmberTools19 [67], which were subsequently converted to GROMACS format using ACPYPE [76]. Five different replicate simulations were built for each system. In each replicate, one copy of each compound was manually placed in different positions around *k-RAS*. These systems were then initially solvated using TIP3P water molecules [77,78] in a dodecahedric box, and the overall system charge was neutralized by randomly replacing 17 water molecules with K+ ions, via the gmx genion tool. Next, the systems underwent a 2-step energy minimization procedure using the steepest descent algorithm [79]: first, with no constraints, and second with hydrogen bond constraints. Velocities were then generated according to a Maxwell distribution at 298.15 K. 

Each replicate was then simulated for 750 ns, using the particle mesh Ewald (PME) method to treat long-range electrostatic interactions [80,81] with a Fourier grid spacing of 0.12 nm and a cutoff of 1.4 nm for direct contributions. Lennard–Jones interactions were calculated using a neighbor pair list with a cutoff of 1.4 nm and using a Verlet scheme [82]. Solute bonds were constrained using the parallel linear constraint solver P-LINCS [83], while water molecules were constrained using the SETTLE algorithm [84]. The temperature was kept at 298.15 K using a Nosé–Hoover thermostat [85,86] with a coupling constant of 1 ps, and an isotropic Parrinello–Rahman barostat [87,88] was used to keep the pressure constant at 1 bar with a coupling constant of 5 ps and a compressibility of 4.5 × 10^−5^ bar^−1^.

Analyses were performed using several GROMACS tools. In addition, plots were made using Gnuplot [89] and all 3D conformations were built using PyMOL [90].

#### 3.5.3. MM/PBSA Calculations

To calculate the binding free energies of the different compounds to the *k-RAS*, we have used the program g_mmpbsa developed by Kumari et al. [91,92]. Analogously to other studies [93], we have used a single trajectory approach which assumes the quadruplex and the ligand conformation in the bound and unbound states to be identical. Equations used to determine binding free energies can be found in the Supplementary Material.

## 4. Conclusions

Bis-quinoline dicarboxamides, particularly those *N*-methylated, are known to be potent G4 stabilizers. In the past, the influence of the central module on compounds capacity to bind and stabilize DNA G4s was well studied [24], but in all these studies only three linking positions of the carboxamide group to quinoline ring were explored: positions 2 (pyridostatin and analogs) [94], 3 (e.g., 360A) and 6 (e.g., 307A) [15]. In this work, we synthesized new bis-quinolinyl/isoquinolinyl-2,6-pyridine dicarboxamides to study the influence of the position of carboxamide linker in the quinoline/isoquinoline rings on the molecular geometry and electronic distribution, and how these parameters impact on compounds’ selective binding and stabilization of G4s of different topologies.

Following a simple synthetic protocol, we were able to successfully obtain six bis-quinolinyl and two bis-isoquinolinyl derivatives, compounds **1a–h**, with yields of 50–94% (Scheme 1). FRET melting experiments revealed that, from these, only compounds with the amide-NH linked to positions 6 (**1d**) and 4 (**1e**), that is, with a relative 1,4-position between this group and the quinoline nitrogen, showed moderate G4 stabilizing capacity, with a preference for the hybrid h-Telo G4. Similarly, it was shown that the presence of a 4-amine group in the quinoline ring of *N*^2^,*N*^6^-di(quinolin-6-yl)pyridine-2,6-dicarboxamide leads to a G4 ligand as potent as the methylated counterpart [15]. This may be explained by an increased fraction of protonated quinoline nitrogens, due to the electron donator character of amine/amide-NH in a 1,4-relative position. Upon methylation of the quinoline/isoquinoline nitrogens, all compounds became much better G4 stabilizers (**1c** vs. **2a**, **1e** vs. **2c**, **1g** vs. **2d** and **1h** vs. **2b**), showing a relative potency trend of **2a** > **2d** > **2c** > **2b**, as determined by FRET and CD-melting assays, and a general preference for parallel G4 structures. In addition, FRET-melting competition assays showed that ligands **2a**–**2d** are selective to G4s up to around 100-fold concentration of double-stranded DNA. We have also studied by CD the capacity of **2a**–**2d** to induce a G4 topology switch and block polymerase activity in a PCR-stop assay. In the conditions of the assay, compound **2a** was able to switch the hybrid 21-nt h-Telo G4 to a parallel topology, whereas **2d** induced an interconversion of this G4 from hybrid to an antiparallel topology. Both compounds were also able to inhibit DNA-polymerization of the primer with the G4-forming sequence present in *c-MYC* promoter, being **2d** more selective, since **2a** also inhibited the polymerization of the mutated sequence in G-tract 3.

To understand the molecular features of compounds **2a**–**2d** behind the observed significant differences between these analogs on G4 stabilization, we performed molecular dynamic simulations using the parallel k-RAS22 G4 structure determined by NMR (PDB ID 5i2v). Figure 6 shows that the best G4 stabilizers **2a**, **2c** and **2d** bind to the G-quartet through π-π stacking between the quinoline/isoquinoline and pyridine rings of the ligand and the guanines of the G4, as well as through favorable electrostatic interactions between the cationic quinoline/isoquinoline nitrogens and the G4 phosphate backbone. Moreover, the position of the methyl groups in relation to the pyridine-2,6-dicarboxamide central module is determinant for the overall geometry of the ligand. The best G4 stabilizer (**2a**) can adopt an almost planar configuration, which optimizes the π-π stacking interactions, whereas the worst G4 stabilizer (the isoquinoline derivative **2b**) interacts preferentially with the groove of a loop of the G4, in a complete twisted configuration.

Quadruplex-interactive small molecules have a wide potential application, not only as drugs but also as sensors of quadruplex structures. With this work we increased the portfolio of G4 ligands of the pyridine-2,6-dicarboxamide family and showed that introduction of an electron donator group in position 4 of que quinoline ring increases G4 stabilization capacity of the ligands, whereas, upon *N*-methylation of the quinoline or isoquinoline rings, the relative spatial orientation of the two quinoline/isoquinoline rings determines the ligands mode and strength of binding to G4s. Overall, we report here new isomers of the potent G4 ligand 360A (**2a**) presenting different strengths and modes of binding to G4s, which may be important when seeking for selectivity. This was here shown by the new bis-methyl-isoquinolinium derivative **2d** which is a less potent G4 stabilizer than **2a** but is more selective than the latter one in blocking DNA polymerization upon stabilization of the 2-3-4-5 G4 loop-isomer of the *c-MYC* promoter sequence (Figure 3). Knowing the structural features of these molecules governing the binding to G4 structures is crucial for the rational development of better therapeutics and more selective G4 sensors.

## Figures and Tables

**Table 1:** FRET-Melting ΔT_m_ values for *k-RAS* G4 and h-Telo G4 at 0.2 µM FAM/TAMRA-labelled DNA concentration, in presence of the non-methylated dicarboxamide derivatives.

		FRET ΔT_m_ (°C) ^a^							
Ligand (5.0 μM/25 eq)		1a	1b	1c	1d	1e	1f	1g	1h
*Sequence*	*k-RAS*	<0.5	1.0 ± 0.2	3.0 ± 0.5	8.0 ± 0.2	8.0 ± 0.1	1.8 ± 0.2	<0.5	<0.5
	h-Telo	<0.5	1.8 ± 0.2	2.8 ± 0.4	18.0 ± 0.3	11.8 ± 0.2	4.0 ± 0.3	<0.5	<0.5

^a^ Experiments performed in cacodylate buffer 60 mM K^+^, pH 7.4. DNA G4 melting: *k-RAS* = 49.0 ± 0.2 °C; h-Telo = 56.9 ± 0.2 °C

**Table 2:** Melting temperature variations (ΔT_m_) values for the tested sequences in the presence of the methylated dicarboxamide derivatives, obtained by FRET-melting assay.

FRET ΔT_m_ (°C) ^a^													
Ligand (eq)		2a			2b			2c			2d		
		0.5	2.5	5	5	12	25	5	12	25	5	12	25
*Sequence*	*k-RAS*	14.0 ± 0.3	32.8 ± 0.2	35.6 ± 0.3	4.0 ± 0.2	9.0 ± 0.1	11.1 ± 0.2	11.8 ± 0.2	16.6 ± 0.2	18.2 ± 0.2	15.0 ± 0.2	23.1 ± 0.1	26.1 ± 0.2
	h-Telo	1.1 ± 0.2	13.2 ± 0.2	18.8 ± 0.3	2.3 ± 0.1	8.0 ± 0.3	10.1 ± 0.1	9.8 ± 0.2	13.1 ± 0.1	14.7 ± 0.1	15.1 ± 0.1	20.1 ± 0.2	22.7 ± 0.2
	t-Loop	2.0 ± 0.1	5.6 ± 0.1	6.5 ± 0.1	<0.5	<0.5	1.8 ± 0.2	<0.5	0.7 ± 0.1	1.2 ± 0.1	<0.5	1.5 ± 0.1	2.5 ± 0.1

^a^ Experiments performed in 60 mM K^+^ cacodylate buffer, pH 7.4 with 0.2 µM of oligonucleotide. G4 DNA melting: *k-RAS* = 49.0 ± 0.2 °C; h-Telo = 56.9 ± 0.2 °C; t-Loop = 53.2 ± 1.0 °C.

**Table 3:** Melting temperature variations (ΔT_m_) values for the tested sequences in the presence of the methylated dicarboxamide derivatives, obtained by CD-melting assay.

CD ΔT_m_ (°C) ^a^																	
Ligand (eq)		2a				2b				2c				2d			
		1.0	5.0	10	25	1.0	5.0	10	25	1.0	5.0	10	25	1.0	5.0	10	25
*Sequence*	*k-RAS*	>40	>40	>40	**P**	3.0 ± 0.2	12.2 ± 0.2	15.1 ± 0.2	**P**	13.8 ± 0.3	35.9 ± 0.1	37.1 ± 0.1	>40	12.6 ± 0.4	>40	>40	>40
	h-Telo	3.5 ± 0.1	12.2 ± 0.3	Topology Change		1.7 ± 0.2	4.4 ± 0.2	6.9 ± 0.2	9.2 ± 0.2	3.3 ± 0.1	9.0 ± 0.1	9.0 ± 0.2	10.8 ± 0.3	6.8 ± 0.2	14.9 ± 0.2	19.8 ± 0.4	27.0 ± 1.3
	*c-MYC*	35.7 ± 1.3	>40	>40	>40	2.7 ± 0.2	5.3 ± 0.1	7.0 ± 0.2	9.7 ± 0.2	8.0 ± 0.2	24.1 ± 0.1	25.0 ± 0.1	29.4 ± 0.1	11.8 ± 0.2	31.2 ± 0.2	34.4 ± 0.2	>40

^a^ Experiments performed with 10 µM of oligonucleotide in 20 mM lithium cacodylate buffer supplemented with KCl and LiCl, keeping a constant ionic strenght of 100 mM, pH 7.2, following the CD λ_max_ for each sequence: 265 nm (*k-RAS* and *c-MYC*); 290 nm (h-Telo). DNA melting: *k-RAS* = 48.3 ± 0.2 °C; h-Telo = 59.6 ± 0.9 °C; *c-MYC* = 50.4 ± 1.9 °C. **P** = DNA precipitation.

**Table 4:** Average binding free energy of the representative configurations of the different compounds with the *k-RAS* G4 (PDB ID: PDB 5i2v). Fifty snapshots were used in the calculations.

Ligand	Average Binding Free Energy (kJ/mol)	Van der Waals Energy (kJ/mol)	Electrostatic Energy (kJ/mol)	Polar Solvation Energy (kJ/mol)	SASA Energy (kJ/mol)
**2a**	−207 ± 8	−240 ± 6	−560 ± 4	612 ± 13	−19 ± 1
**2b**	−215 ± 14	−220 ± 5	−518 ± 4	543 ± 17	−19 ± 1
**2c**	−171 ± 8	−248 ± 8	−553 ± 4	649 ± 9	−19 ± 1
**2d**	−203 ± 7	−217 ± 7	−521 ± 5	551 ± 14	−17 ± 1

## Data Availability

Data is contained within the article and supplementary material.

## Supplementary Materials

The following are available online at https://www.mdpi.com/article/10.3390/ph14070669/s1, Figure S1: FRET-Melting curves of h-Telo (A, blue curve) and k-RAS (B, blue curve) G4 in presence of compound **1e** (red curves) and **1d** (green curves) at 5 µM concentration; Figure S2: FRET-Melting curves of h-Telo G4 (blue curves), in presence of increasing concentration of compounds **2a** (A), **2b** (B), **2c** (C) and **2d** (D); Figure S3: FRET-Melting curves of *k-RAS* G4 (blue curves), in presence of increasing concentration of compounds **2a** (A), **2b** (B), **2c** (C) and **2d** (D); Figure S4: FRET-Melting curves of double-stranded t-Loop (blue curves), in presence of increasing concentration of compounds **2a** (A), **2b** (B), **2c** (C) and **2d** (D); Figure S5: CD titration of *c-MYC* G4 in presence of increasing equivalents (0–25) of compound **2a** (A), **2b** (B), **2c** (C), **2d** (D); Figure S6: CD titration of *k-RAS* G4 in presence of increasing equivalents (0–25) of compound **2a** (A), **2b** (B), **2c** (C), **2d** (D); Figure S7: CD titration of *c-MYC* Pu27Mut in presence of increasing equivalents of compound 2a, performed in ThermoPol buffer (20 mM Tris-HCl, 10 mM (NH_4_)_2_SO_4_, 10 mM KCl, 2 mM MgSO_4_, 0.1% Triton^®^ X100, pH 8.8); Figure S8: (A) Results of PCR-stop assay with compounds **1a**–**e** at 50 µM and *c-MYC* gene promoter Pu27. (B) Results of PCR-stop assay with compound **2a** and *c-MYC* gene promoter Pu27 or mutated *c-MYC* gene promoter (Pu27mut); Figure S9: Root mean square deviation (RMSD) of the complex *k-RAS* + ligand, throughout the simulation time for each replicate simulation; Figure S10: Variation of the MM/PBSA binding free energy between the different ligands and *k-RAS* for each replicate simulation; Figure S11: Minimum distance of compound **2a** to each pair of bases of *k-RAS* for all replicate simulations; Figure S12: Minimum distance of compound **2b** to each pair of bases of *k-RAS* for all replicate simulations; Figure S13: Minimum distance of compound **2c** to each pair of bases of *k-RAS* for all replicate simulations; Figure S14: Minimum distance of compound **2d** to each pair of bases of *k-RAS* for all replicate simulations. Figures S15–S25: NMR spectra of the synthesized molecules; Figures S26–S37: TIC chromatogram of samples with peak integrations and mass spectra. Table S1: Sequences used in FRET-melting experiments; Table S2: Sequences used in CD experiments; Table S3: Sequences used in PCR-stop assay.
