# Supplementary material for: New (Iso)quinolinyl-pyridine-2,6-dicarboxamide G-Quadruplex Stabilizers. A Structure-Activity Relationship Study
Source: Pharmaceuticals (Basel). 2021 Jul 13;14(7):669. doi: 10.3390/ph14070669 (PMC8308870; doi:10.3390/ph14070669)
Supplement: Supplementary file 1 [file pharmaceuticals-14-00669-s001.zip › pharmaceuticals-1260011-SI.pdf]

# New (Iso)Quinolinyl-Pyridine-2,6-dicarboxamide G-Quadruplex Stabilizers. A Structure-Activity Relationship Study

Enrico Cadoni [1], Pedro R. Magalhães [2], Rita Emídio [2], Eduarda Mendes [1,3], Jorge Vítor [4], Josué Carvalho [5], Carla Cruz [5], Bruno L. Victor [2] and Alexandra Paulo [1,3,*]

[1] Research Institute for Medicines (iMed.ULisboa), Faculty of Pharmacy, Universidade de Lisboa, Av. Prof. Gama Pinto, 1649-003 Lisboa, Portugal;

[2] BioISI - Biosystems & Integrative Sciences Institute, Faculty of Sciences, University of Lisboa, Campo Grande, C8 bdg, 1749-016 Lisboa, Portugal

[3] Dep. of Pharmaceutical Sciences and Medicines, Faculty of Pharmacy, Universidade de Lisboa, Av. Prof. Gama Pinto, 1649-003 Lisboa, Portugal;

[4] Dep. of Pharmacy, Pharmacology and Health Technologies, Faculty of Pharmacy, Universidade de Lisboa, Av. Prof. Gama Pinto, 1649-003 Lisboa, Portugal;

[5] CICS-UBI - Centro de Investigação em Ciências da Saúde, Universidade da Beira Interior, Av. Infante D. Henrique, 6200-506, Covilhã, Portugal

*Correspondence: mapaulo@ff.ulisboa.pt;

**Supplementary materials**

1. General ........ 1
2. FRET melting experiments ........ 2
3. CD Titrations ........ 4
4. PCR-Stop assay ........ 7
5. MM/PBSA calculations ........ 8
6. NMR spectra ........ 15
7. LC-MS Characterization ........ 26
8. Supplemental references ........ 30

## 1. General

All the reagents were purchased from Sigma-Aldrich and Merck and used without further purification.

NMR-Spectra were recorded on a Bruker 300 Ultrashield 300MHz, using 300 MHz scan for $^{1}$H-NMR spectra and 75 MHz for $^{13}$C.

Purity of compounds submitted to biophysical, biochemical or biological tests were in all cases > 90 % as determined by HPLC–MS (HPLC Waters Alliance 2695 coupled to a Photodiode Array Detector Waters 996 PDA and a Triple Quadrupole MS Micromass Quattro Micro API), using a Phenomenex Luna 5u HILIC 200A (100x3mm; 5µm) column and a ACN-HCOOH 0.5% (40:60) elution system at 0.2 ml/min.

## 2. FRET melting experiments

FRET melting assays were performed on a 7300 RT-PCR equipment from Applied Biosystems. The test compound solutions (50 µL) were distributed across 96-well RT-PCR plates (PCR-96-FLT-C, Axygen, Inc.). Experiments were performed in cacodylate buffer, 60 mM $K^+$, pH 7.4, with 0.2 µM of oligonucleotide.

Standard and labelled HPLC-purified oligonucleotides were purchased from STABVIDA (Portugal), and sequences are depicted in table **S1**.

*Table S1. Sequences used in FRET-melting experiments.*

| Name | Sequence | Topology | Tm (°C) |
|---|---|---|---|
| *k-RAS* | 5'-FAM-AGGGCGGTGTGGGAAGAGGGA-TAMRA-3' | Parallel G4 | 49.0 ± 0.2 |
| *h-Telo* | 5'-FAM-GGGTTAGGGTTAGGGTTAGGG-TAMRA-3' | Hybrid G4 | 56.9 ± 0.2 |
| *T-Loop* | 5'-FAM-TATAGCTATATTTTTTTATAGCTATA-TAMRA-3' | dsDNA | 53.2 ± 1.0 |
| 26merA<br>26merB | 5'-CAATCGGATCGAATTCGATCCGATTG-3'<br>5'-GTTAGCCTAGCTTAAGCTAGGCTAAG-3' | Self-hybridizing duplex DNA | |

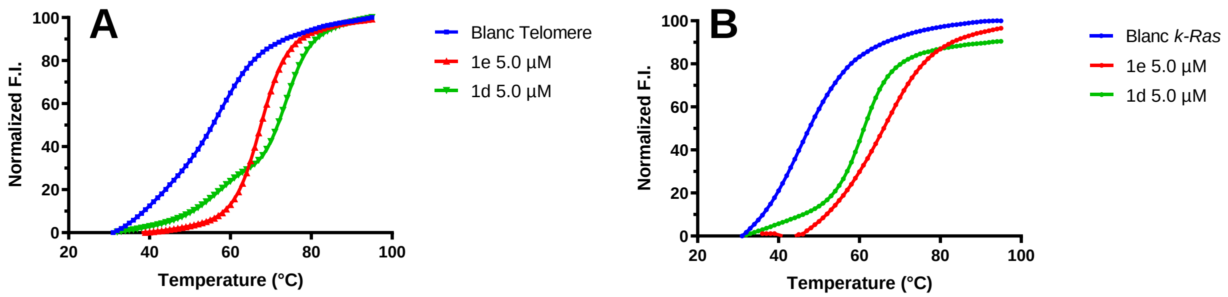


*Figure S1. FRET-Melting curves of h-Telo (A, blue curve) and k-RAS (B, blue curve) in presence of compound* ***1e*** *(red curves) and* ***1d*** *(green curves) at 5 µM concentration (25 eq.).*

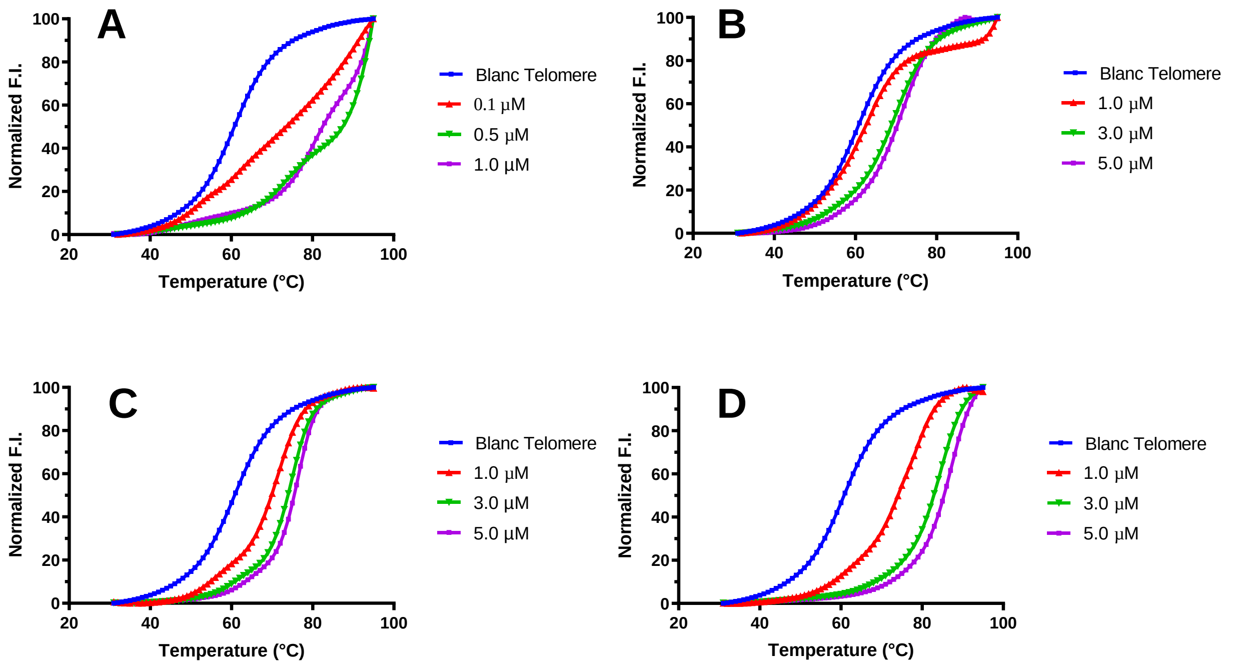


*Figure S2. FRET-Melting curves of h-Telo (blue curves), in presence of increasing concentration of compounds **2a** (A), **2b** (B), **2c** (C) and **2d** (D).*

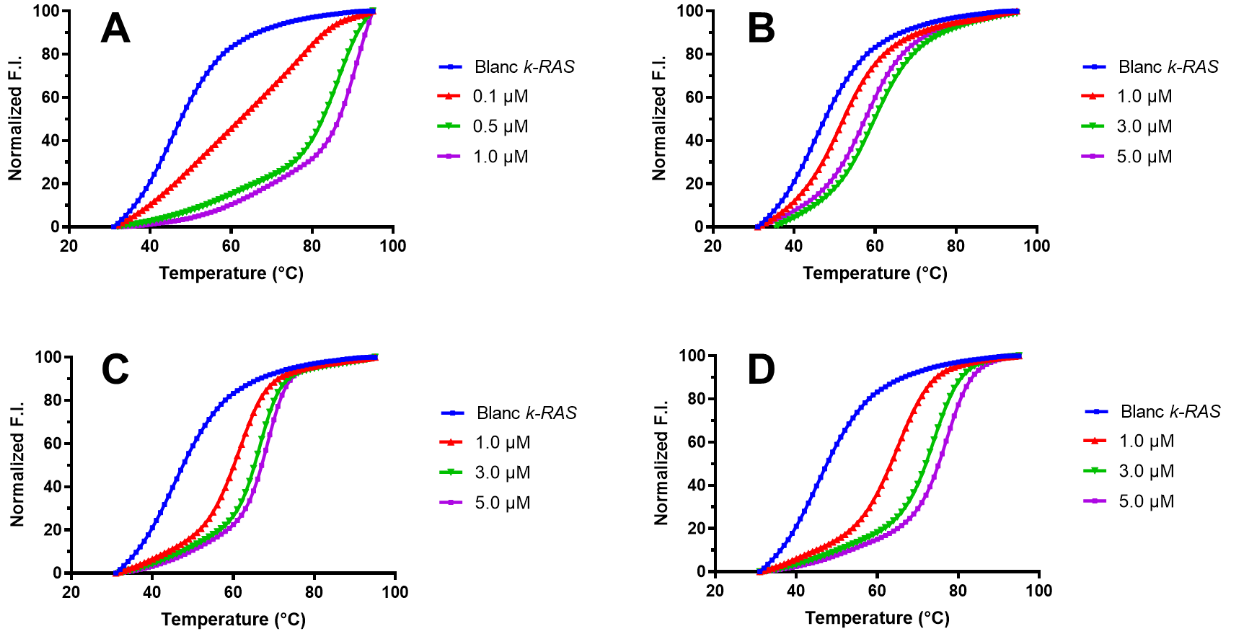


*Figure S3. FRET-Melting curves of k-RAS (blue curves), in presence of increasing concentration of compounds **2a** (A), **2b** (B), **2c** (C) and **2d** (D).*

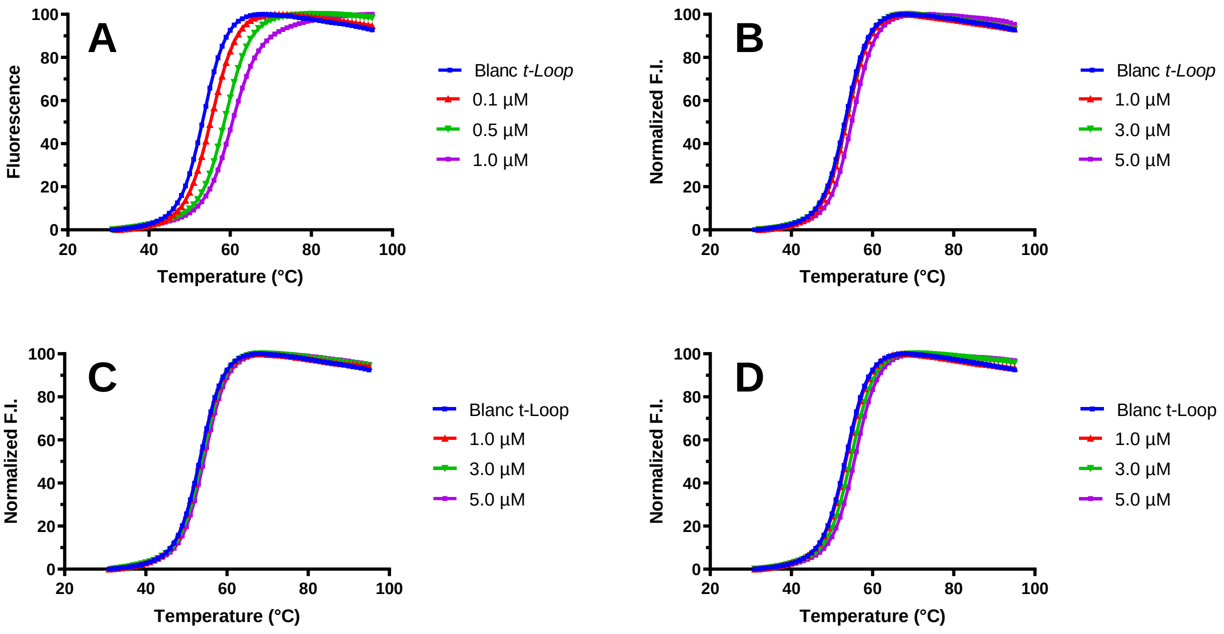


*Figure S4. FRET-Melting curves of double-stranded t-Loop (blue curves), in presence of increasing concentration of compounds **2a** (A), **2b** (B), **2c** (C) and **2d** (D).*

## 3. CD Titrations

CD titrations and melting assays were performed on Jasco J-815 spectropolarimeter equipped with a Peltier-type temperature control system (model CDF-426S/15), using an instrument scanning speed of 200 nm/min with a response time of 1 s in wavelengths ranging from 200 to 340 nm. CD-melting experiments were performed with 10 µM of oligonucleotide in 20 mM lithium cacodylate buffer supplemented with KCl and LiCl as described in Material and Methods (& 4.4), keeping a constant ionic strength of 100 mM. The DNA sequences used for the experiments, and their respective melting temperatures are depicted in table S2.

*Table S2. Sequences used in CD experiments.*

| **Name** | **Sequence** | **Topology** | **Tm (°C)** |
|---|---|---|---|
| *k-RAS* | 5’-AGGGCGGTGTGGGAAGAGGGA- 3’ | Parallel G4 | 48.3 ± 0.2 |
| *H-Telo* | 5’- GGGTTAGGGTTAGGGTTAGGG-3’ | Hybrid G4 | 59.6 ± 0.9 |
| *c-MYC* | 5’-TGGGGAGGGTGGGGAGGGTGGGGAAGG-3’ | Parallel G4 | 50.4 ± 1.9 |

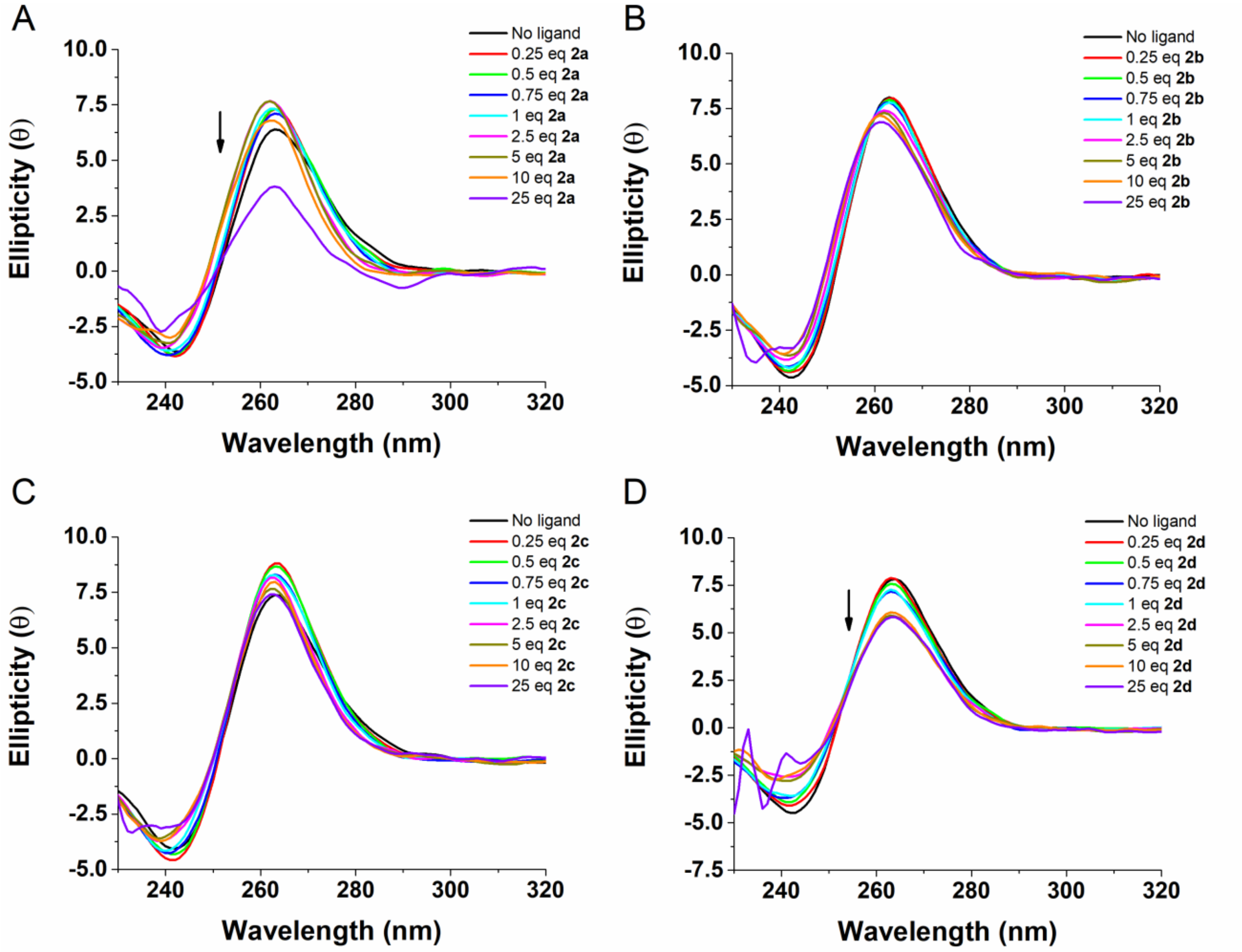


*Figure S5. CD titration of c-MYC in presence of increasing equivalents (0-25) of compound 2a (A), 2b (B), 2c (C), 2d (D), at a concentration of 10 µM, performed in 20 mM lithium cacodylate containing 100 mM LiCl.*

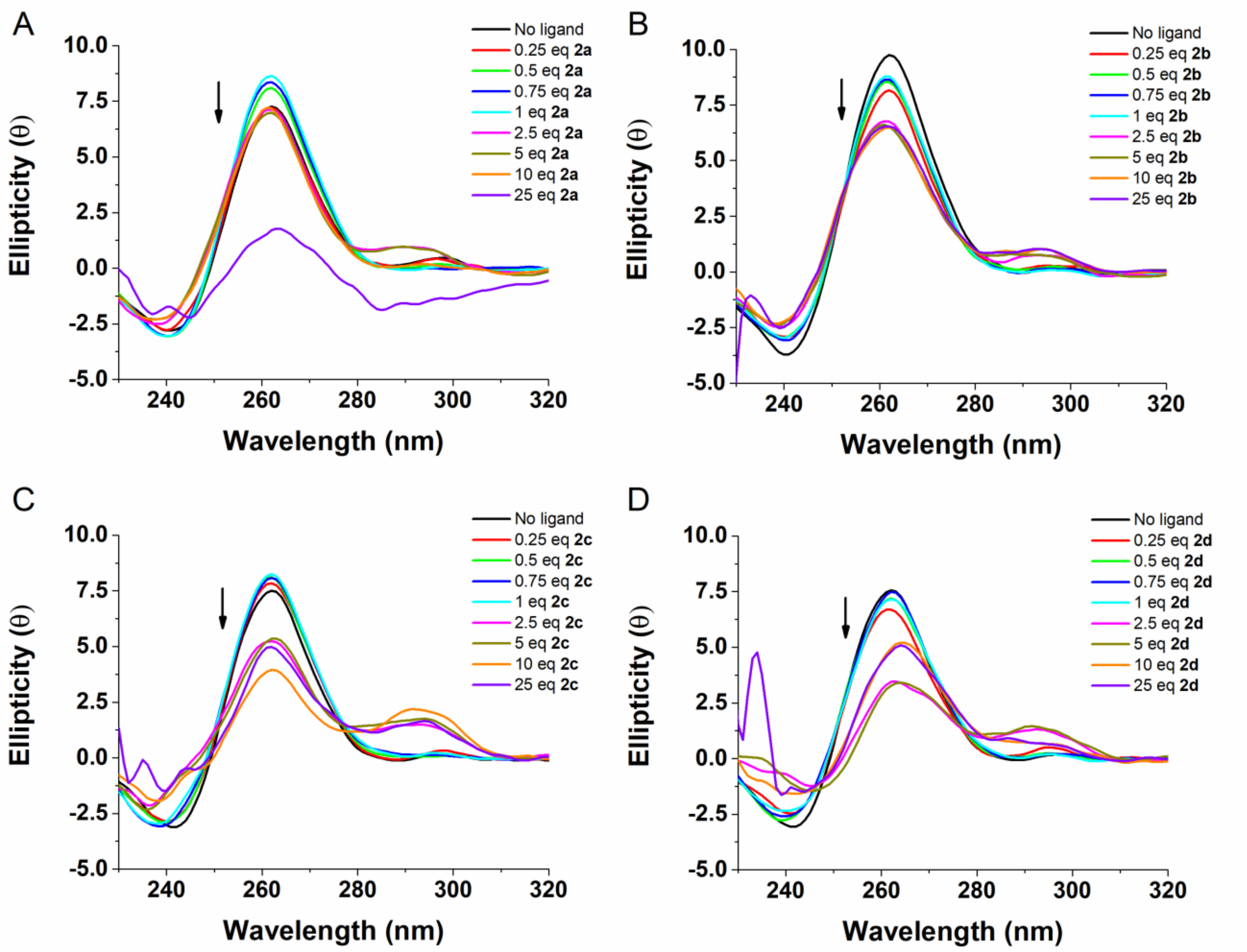


*Figure S6. CD titration of k-RAS in presence of increasing equivalents (0-25) of compound 2a (A), 2b (B), 2c (C), 2d (D), at a concentration of 10 µM, performed in 20 mM lithium cacodylate containing 50 mM KCl and 50 mM LiCl.*

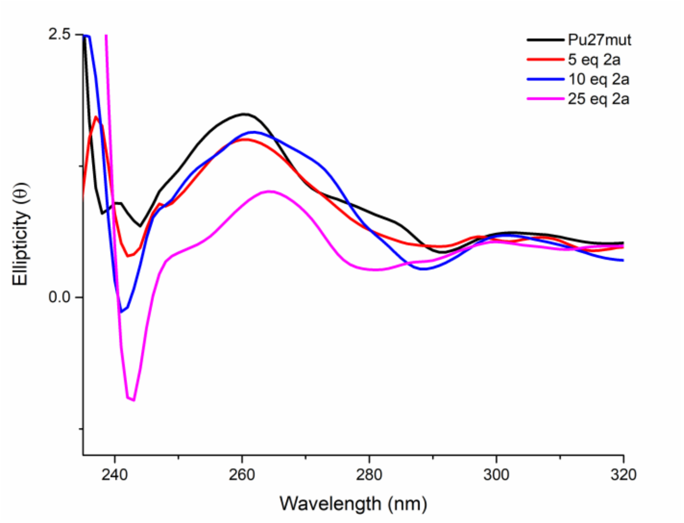


*Figure S7. CD titration of c-MYC Pu27mut (sequence in table S3) in presence of increasing equivalents of compound 2a, performed in ThermoPol buffer (20mM Tris-HCl, 10mM $(NH_4)_2SO_4$, 10mM KCl, 2mM $MgSO_4$, 0.1% Triton® X100, pH 8.8).*

## 4. PCR-Stop assay

The primer sequences used in PCR-stop assay are depicted in table S3. Sequence-design was adapted from [1].

*Table S3. Sequences used in PCR-stop assay*

| Name | Sequence |
|---|---|
| Pu27 | 5’-TGGGGAGGGTGGGGAGGGTGGGGAAGG-3’ |
| Pu27mut | 5’-TGGGGAGGGTGGAAAGGGTGGGGAAGG-3’ |
| Pu27REV | 5’-ATCGAATCGCTTCTCGTCCTTCCCCA-3’ |
| Pu27mut2 | 5’-TGGGGAGGGTGGAAAGGGTTTGGAAGG-3’ |
| Pu27REV2 | 5’-ATCGATCGCTTCTCGTCCTTCCAAA-3’ |

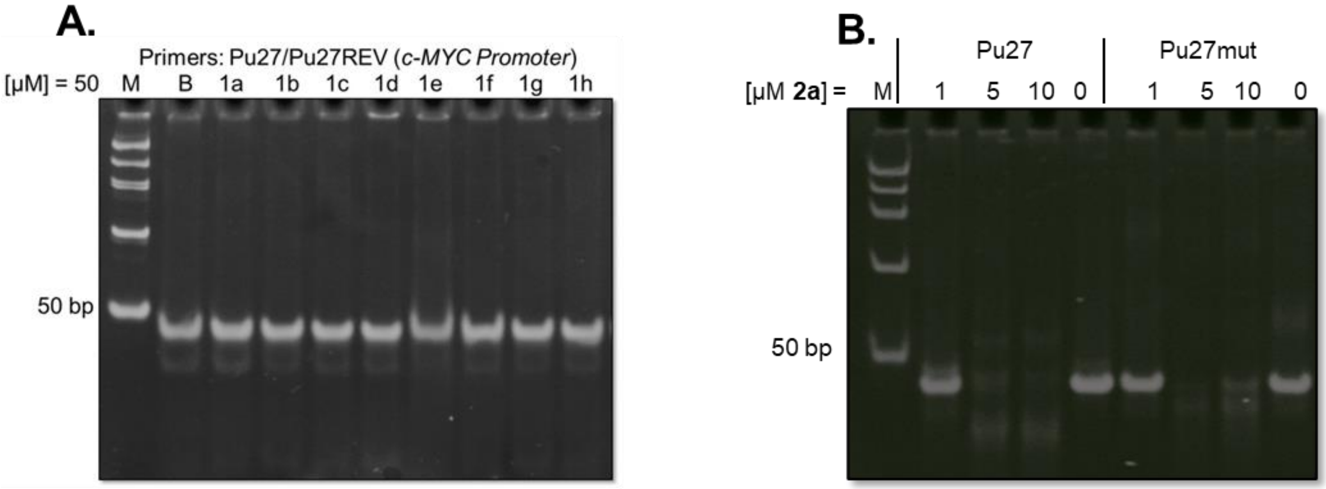


*Figure S8. A) Results of PCR-stop assay with compounds **1a-h** at 50 µM and c-MYC gene promoter Pu27. B) Results of PCR-stop assay with compound **2a*** and c-MYC gene promoter Pu27 or *with mutated c-MYC gene promoter (Pu27mut). In absence of compound, the 43 bp PCR products from constructions with Pu27 is formed.*

## 5. MM/PBSA calculations

The equation used to determine the binding free energy of the ligands to the quadruplex is expressed as:

$$\Delta G_{bind} = \Delta G_{complex}^{vdW} + \Delta G_{complex}^{ele} + \Delta G^{polar} + \Delta G^{nonpolar} \quad \text{(eq 1)}$$

with $V_{complex}$vdW and $V_{complex}$ele being the quadruplex–ligand van der Waals and electrostatic interaction energies, respectively. The polar and the nonpolar contributions are expressed as

$$\Delta G^{polar} = G_{complex}^{polar} - (G_{protein}^{polar} + G_{ligand}^{polar}) \quad \text{(eq 2)}$$

and

$$\Delta G^{nonpolar} = G_{complex}^{nonpolar} - (G_{protein}^{nonpolar} + G_{ligand}^{nonpolar}) \quad \text{(eq 3)}$$

The Poisson–Boltzmann (PB) equation for the polar term is evaluated using the following equation:

$$\nabla \cdot [\varepsilon(r) \nabla \cdot \varphi(r)] - \varepsilon(r) k(r)^2 \sinh[\varphi(r)] + \frac{4\pi\rho^f(r)}{k_B T} = 0 \quad \text{(eq 4)}$$

where ϕ(r) corresponds to the ligand's electrostatic potential, ε(r) the dielectric constant, and $\rho^f(r)$ the fixed charge density. Since the polar solvation energy is known to depend on the chosen value for the dielectric constant $\varepsilon_{solute}$ of the complex [2], several values (2, 4 and 8) were evaluated to inspect its effect on predicted $\Delta G_{bind}$ values. By default, $\varepsilon_{solute}$ is set to 2, but ultimately, we have used a value of 8 which showed to be the most adequate to use for our system. All the other settings in g_mmpbsa were left unaltered (i.e., use of atomic radii as proposed by Bondi [3], linear PB equation solver, 0.05 nm grid resolution, and smoothed van der Waals surface).

To estimate nonpolar solvation energies, we have used a linear dependence of $G_{nonpolar}$ on the SASA, expressed as follows [4]:

$$G^{nonpolar} = \gamma_{surf} SASA + b$$ (eq 5)

where $\gamma_{surf}$ is related to the surface tension of the solvent. A solvent probe radius of 0.14 nm was used to determine the SASA.

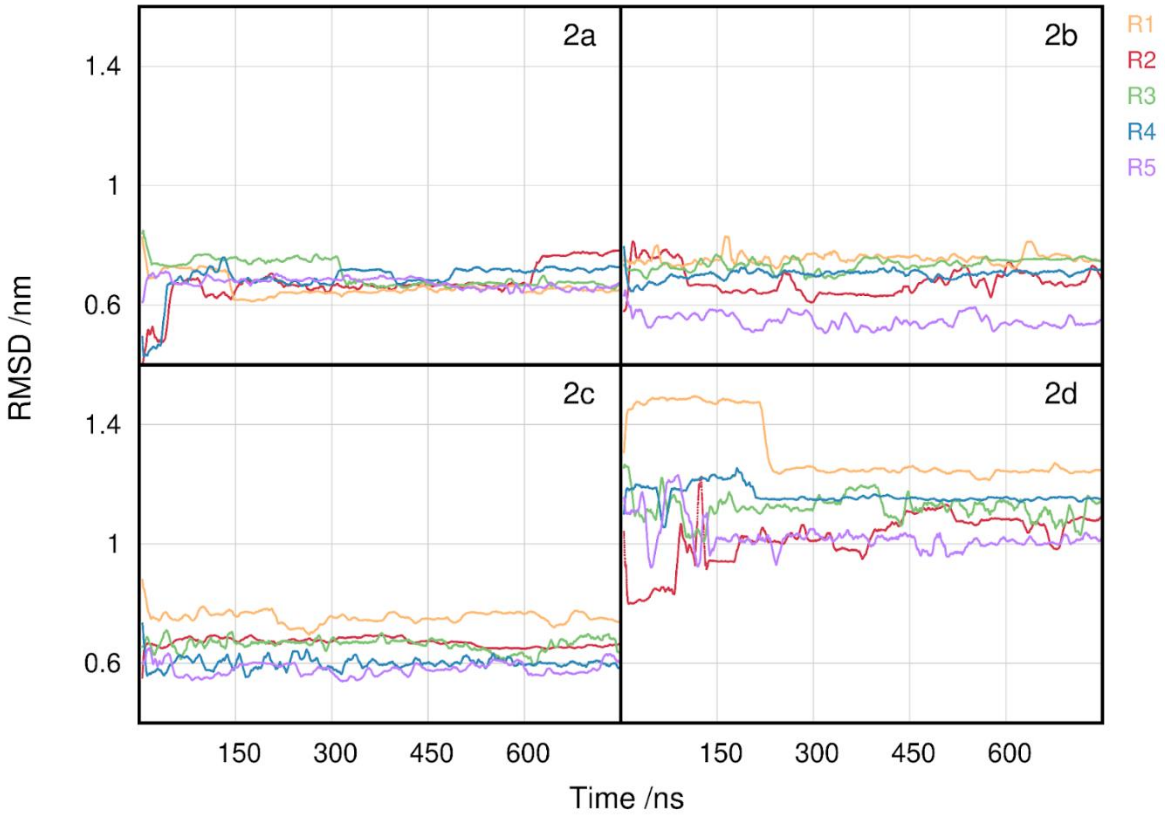


*Figure S9. Root mean square deviation (RMSD) of the complex k-RAS + ligand, throughout the simulation time for each replicate simulation.*

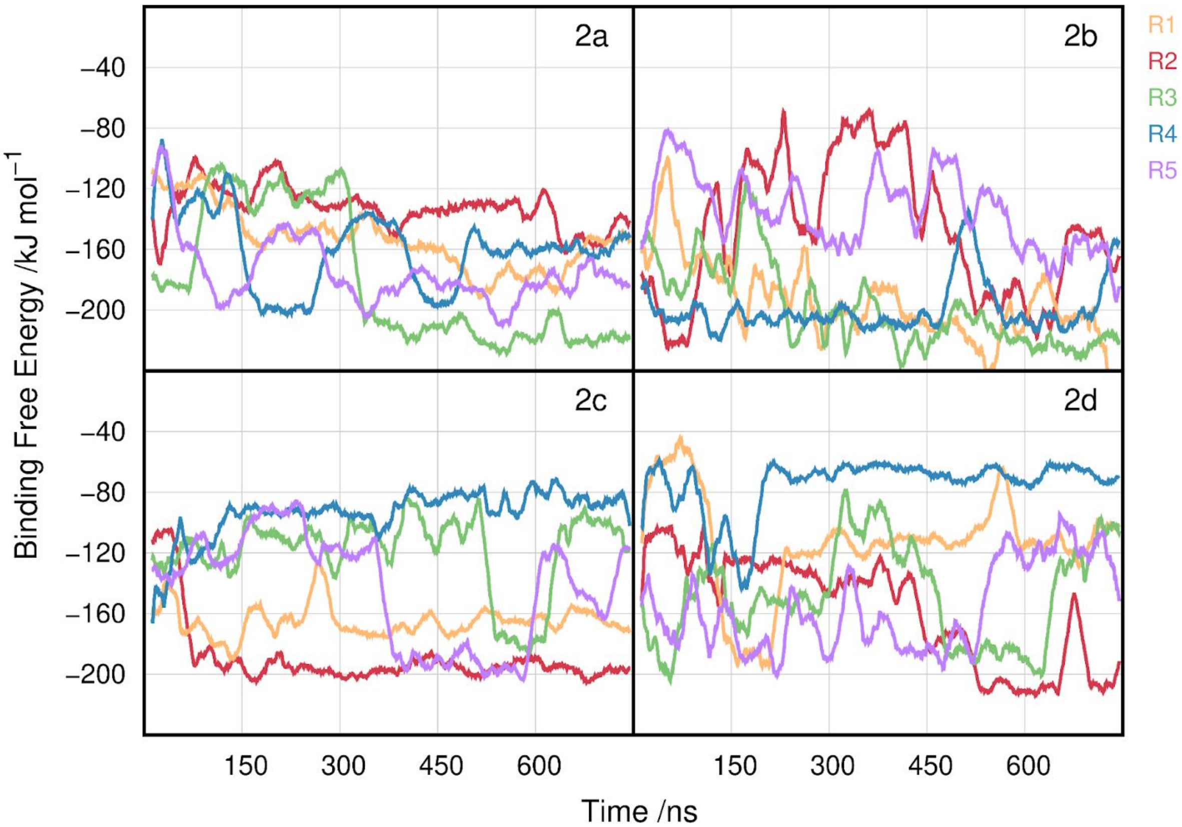


*Figure S10. Variation of the MM/PBSA binding free energy between the different ligands and k-RAS for each replicate simulation.*

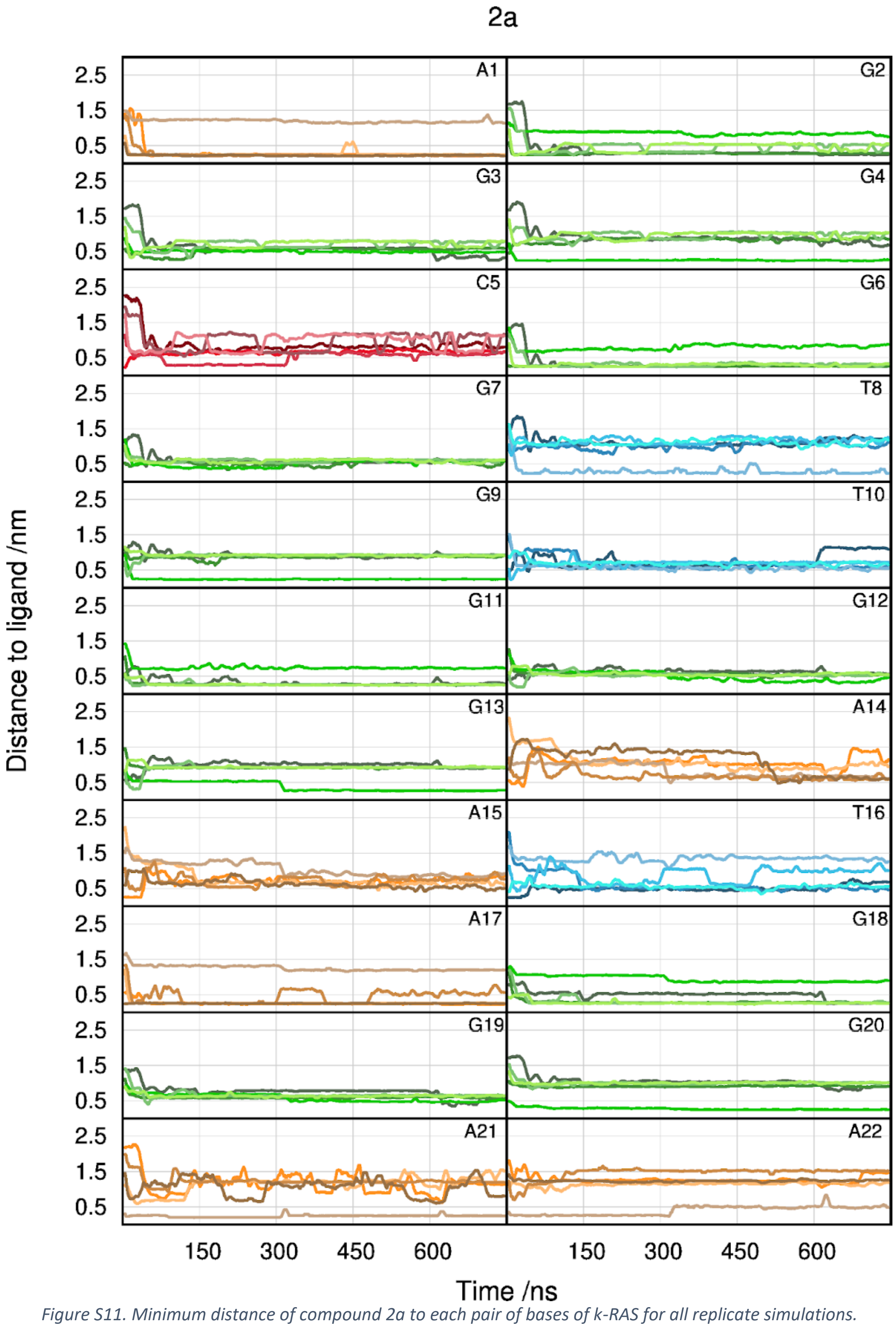

*Figure S11. Minimum distance of compound 2a to each pair of bases of k-RAS for all replicate simulations.*

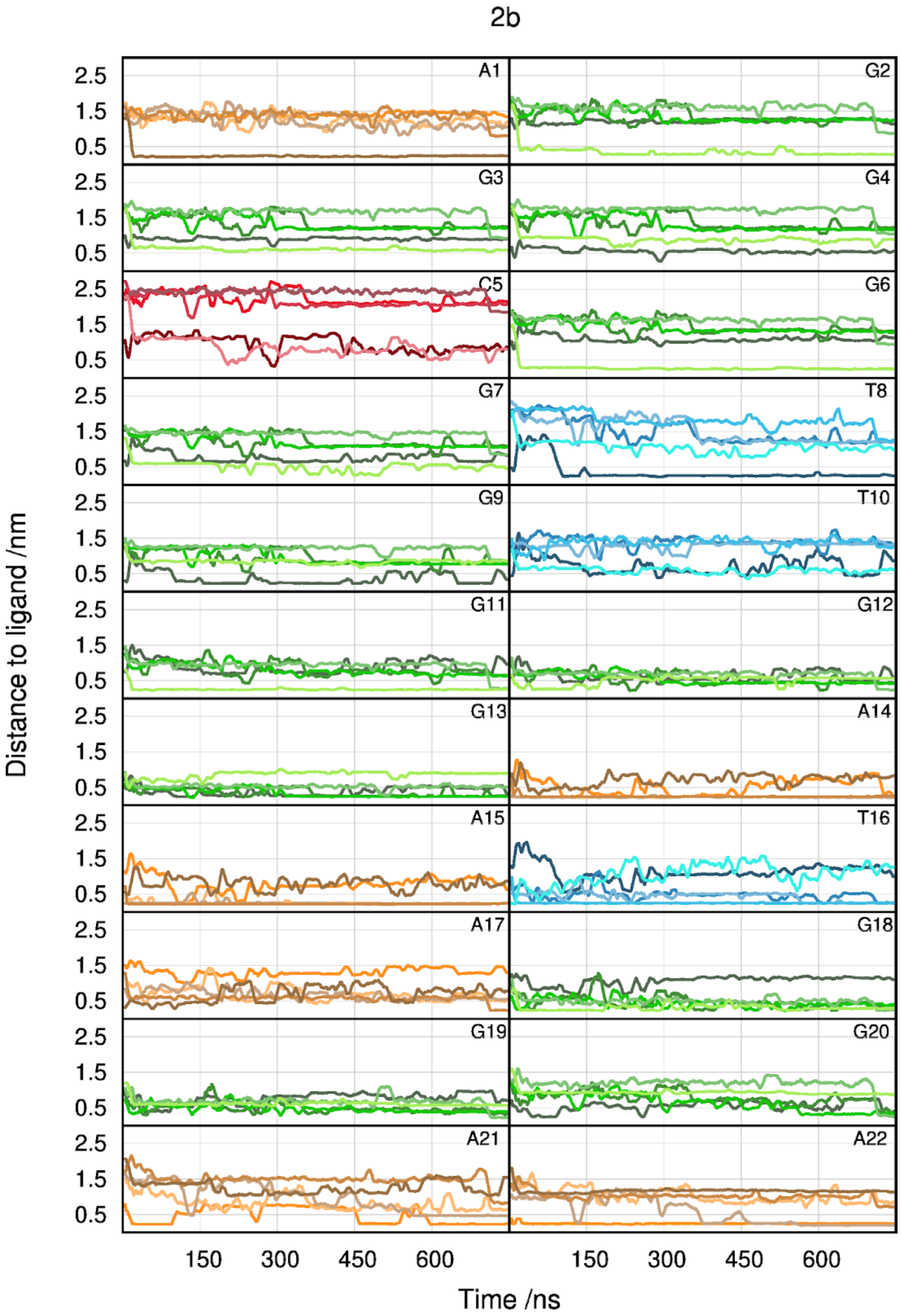

*Figure S12. Minimum distance of compound 2b to each pair of bases of k-RAS for all replicate simulations.*

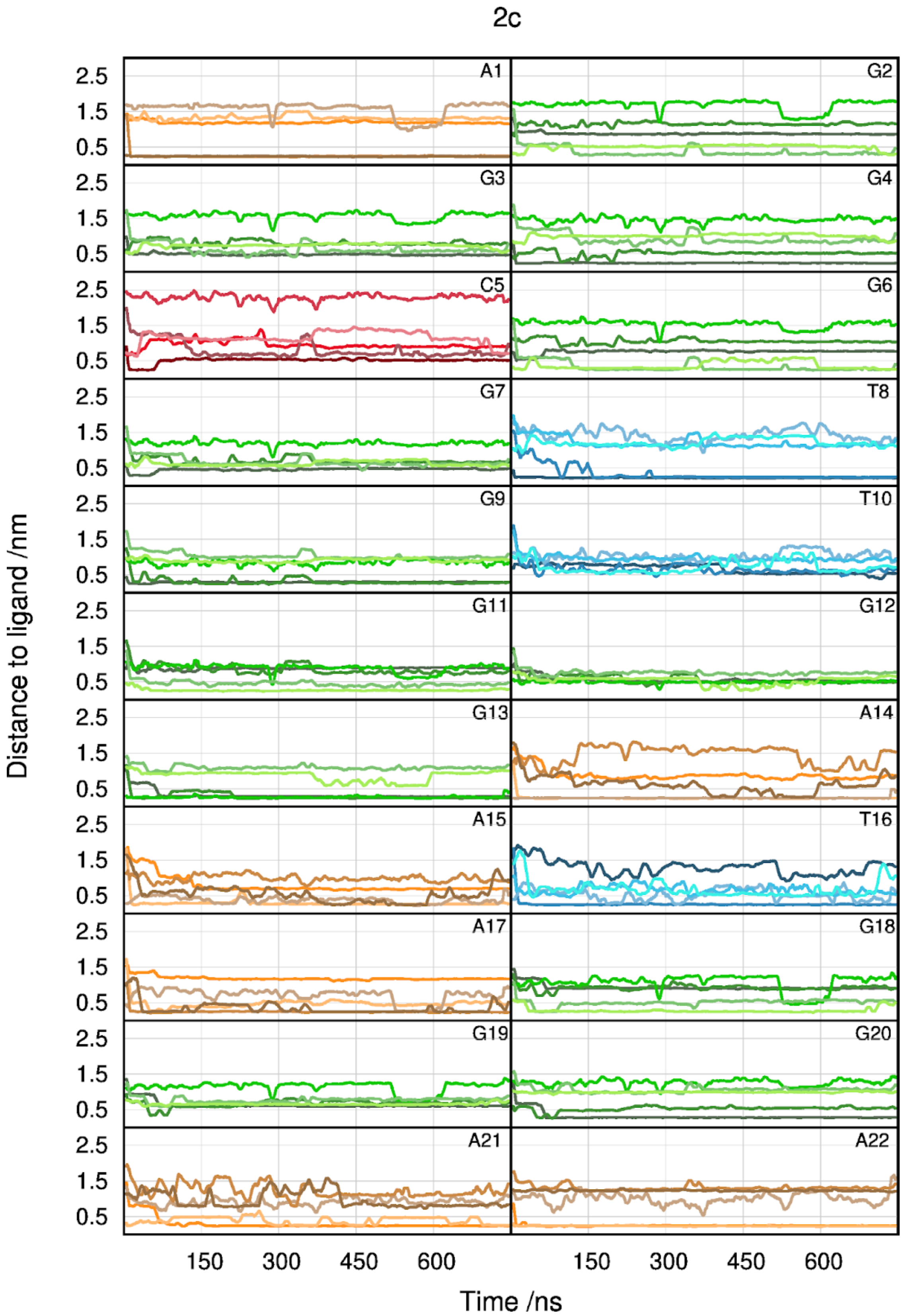

*Figure S13. Minimum distance of compound 2c to each pair of bases of k-RAS for all replicate simulations.*

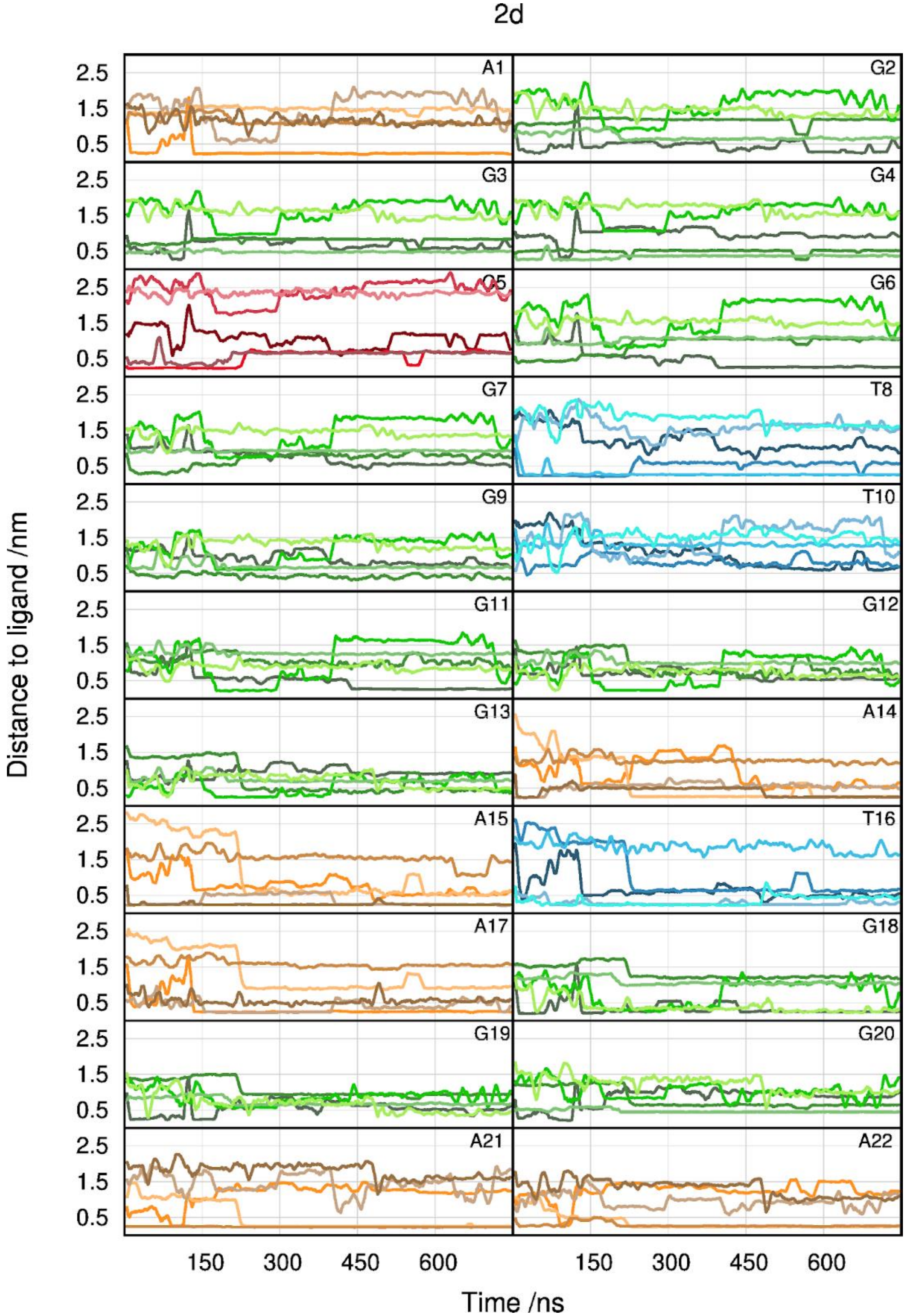


*Figure S14. Minimum distance of compound 2d to each pair of bases of k-RAS for all replicate simulations.*

## 6. NMR spectra

NMR-Spectra were recorded on a Bruker 300 Ultrashield 300MHz, using 300MHz scan for $^{1}$H-NMR spectra and 75 MHz for $^{13}$C.

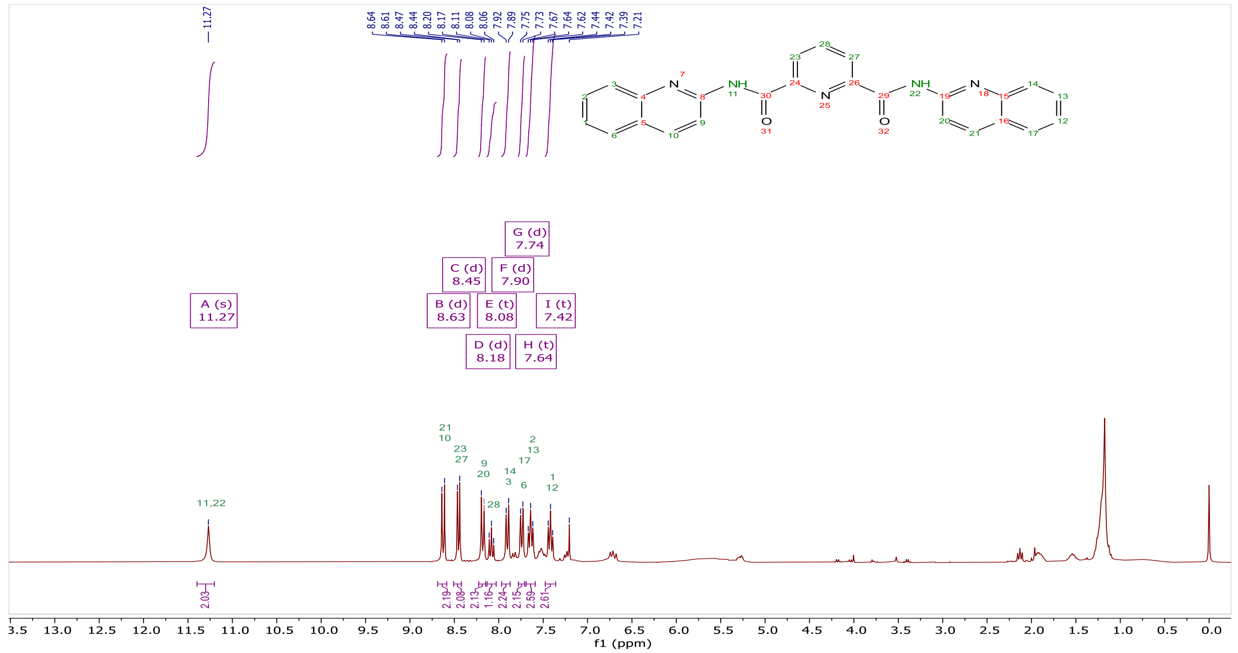

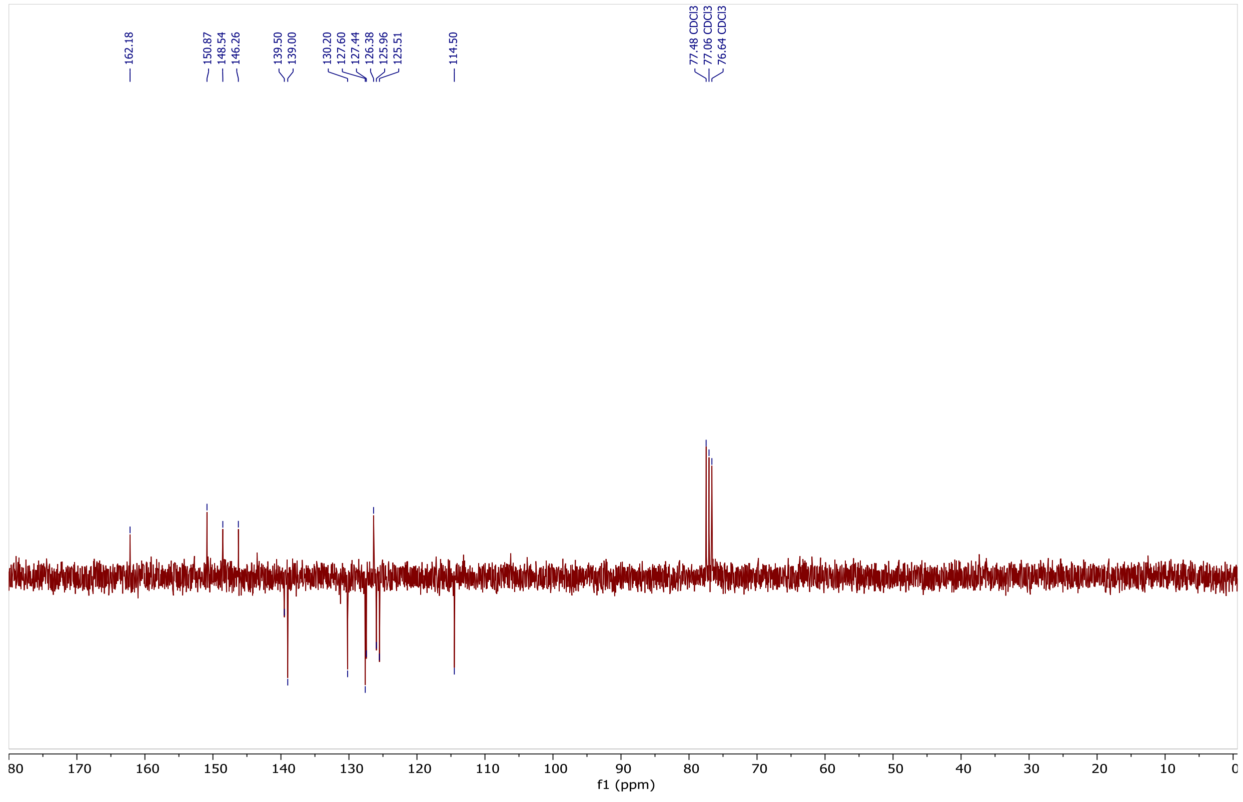

*Figure S15. H-NMR (top) and C-NMR (bottom) spectra of compound **1a**.*

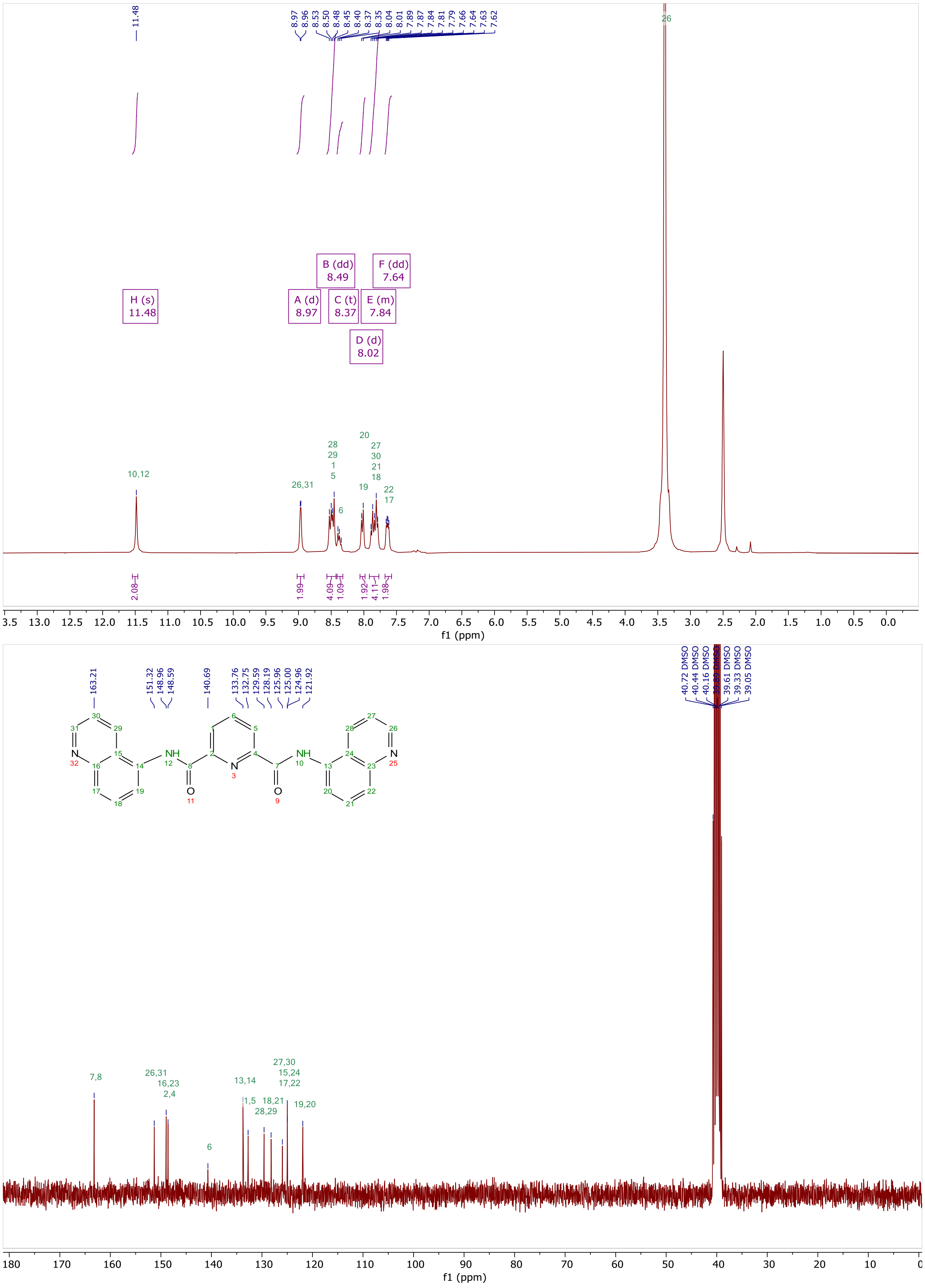


*Figure S16. H-NMR (top) and C-NMR (bottom) spectra of compound* ***1b.***

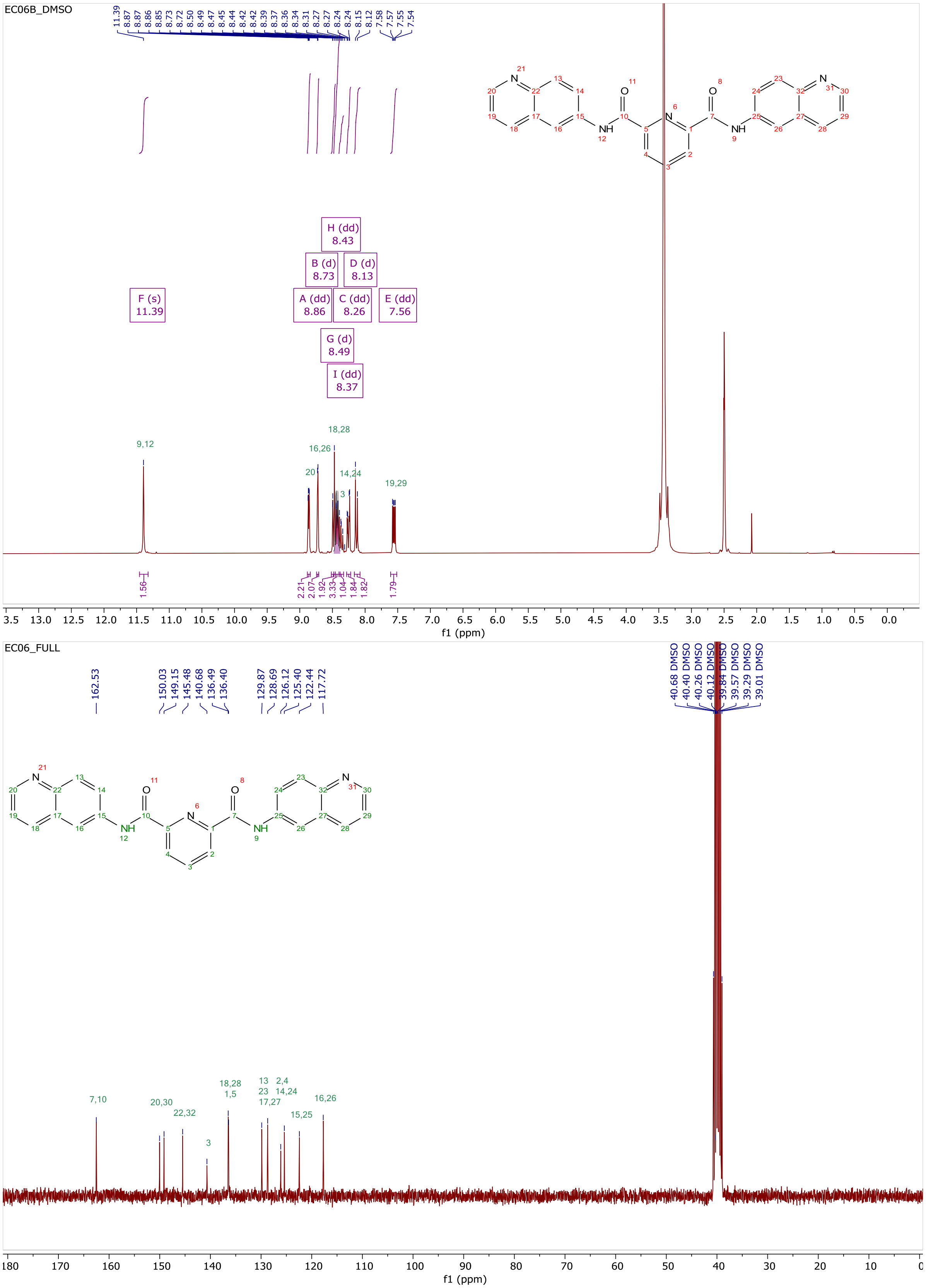


*Figure S17. H-NMR (top) and C-NMR (bottom) spectra of compound* ***1d.***

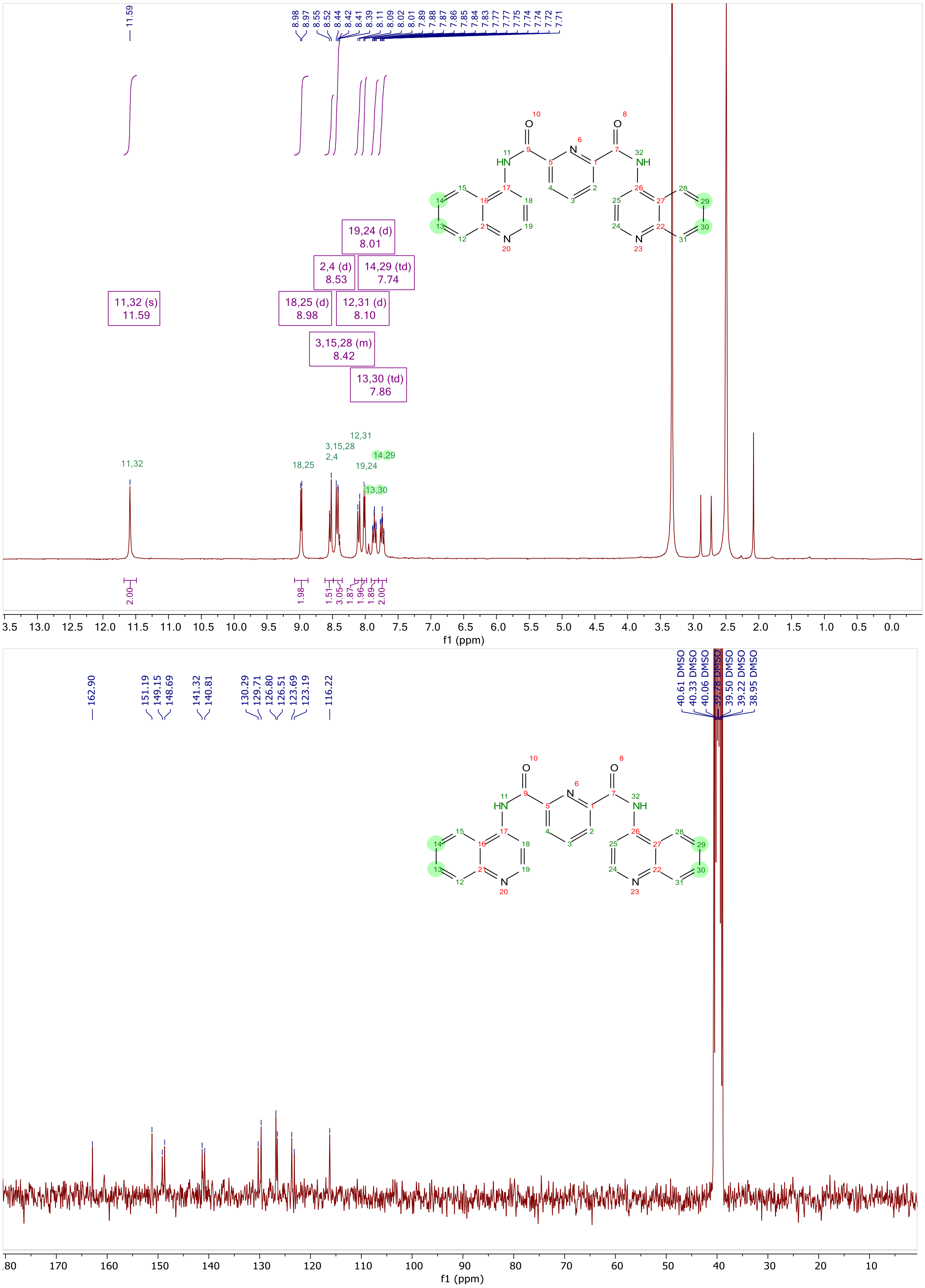


*Figure S18. H-NMR (top) and C-NMR (bottom) spectra of compound **1e**.*

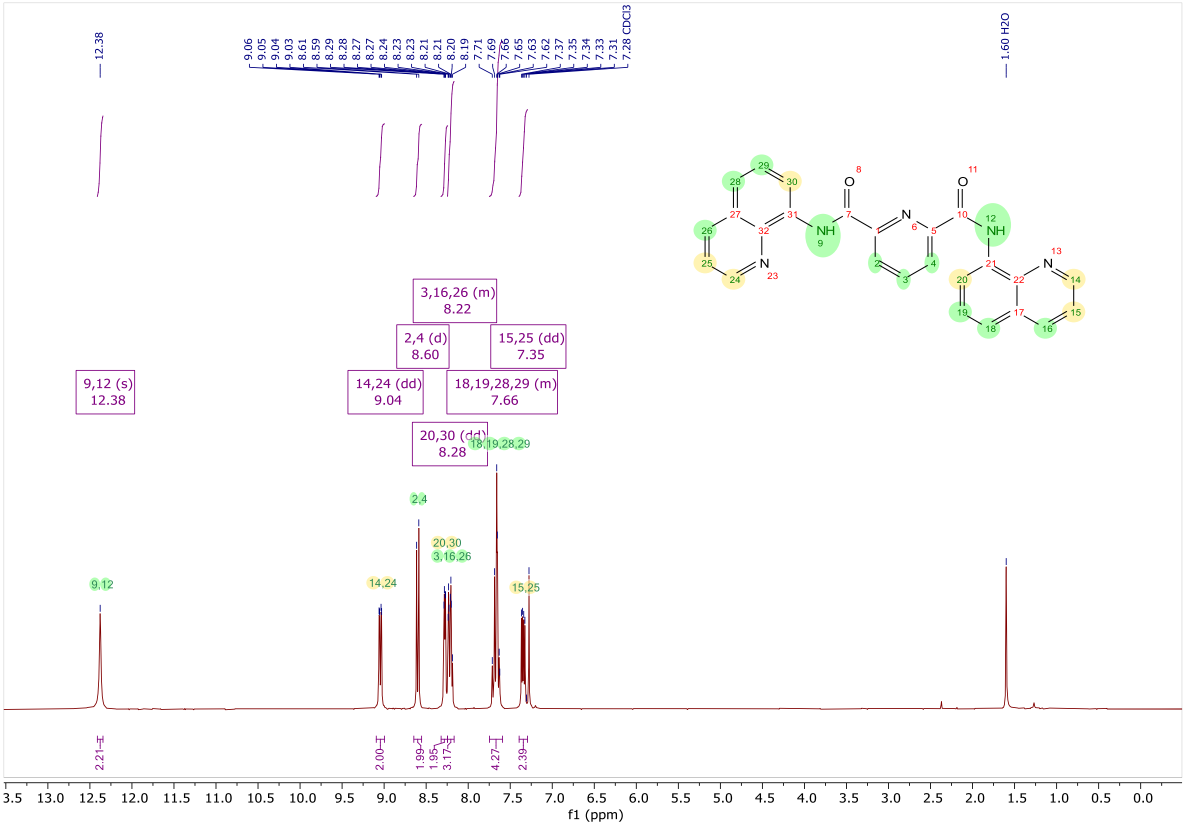


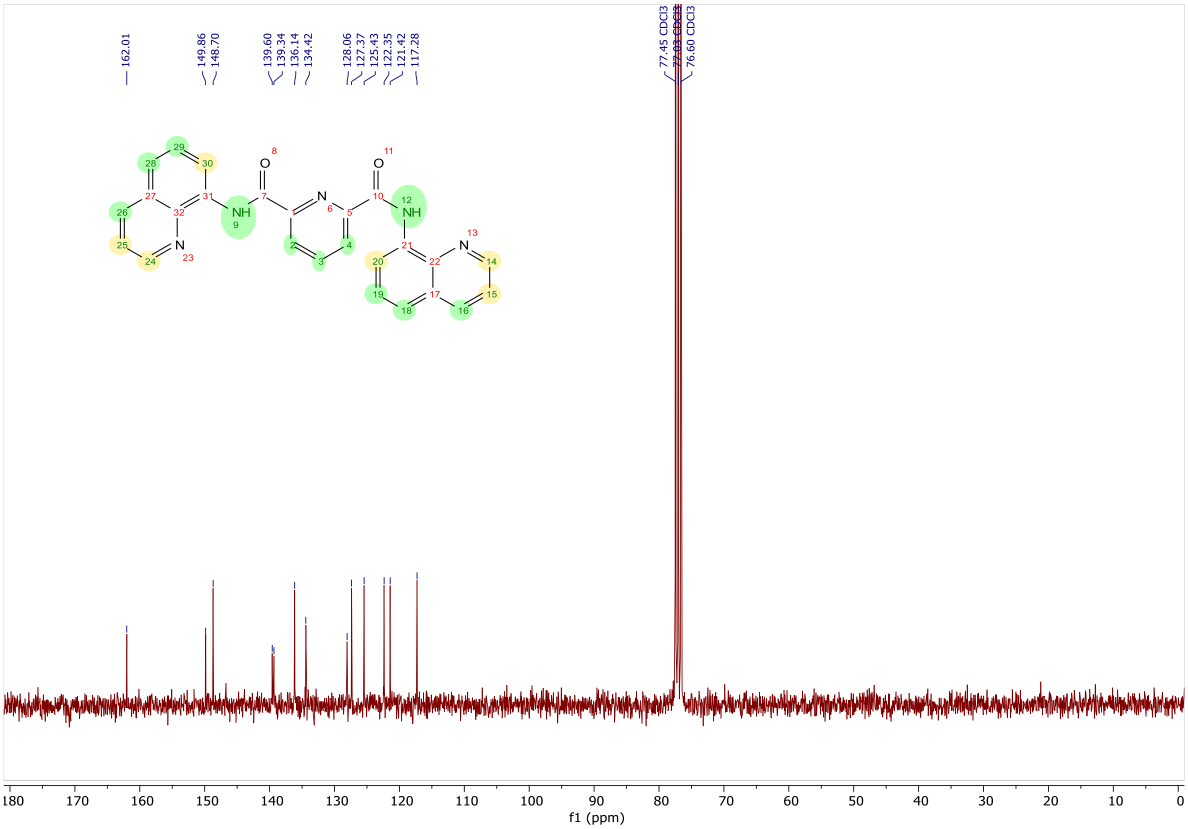


*Figure S19. H-NMR (top) and C-NMR (bottom) spectra of compound **1f**.*

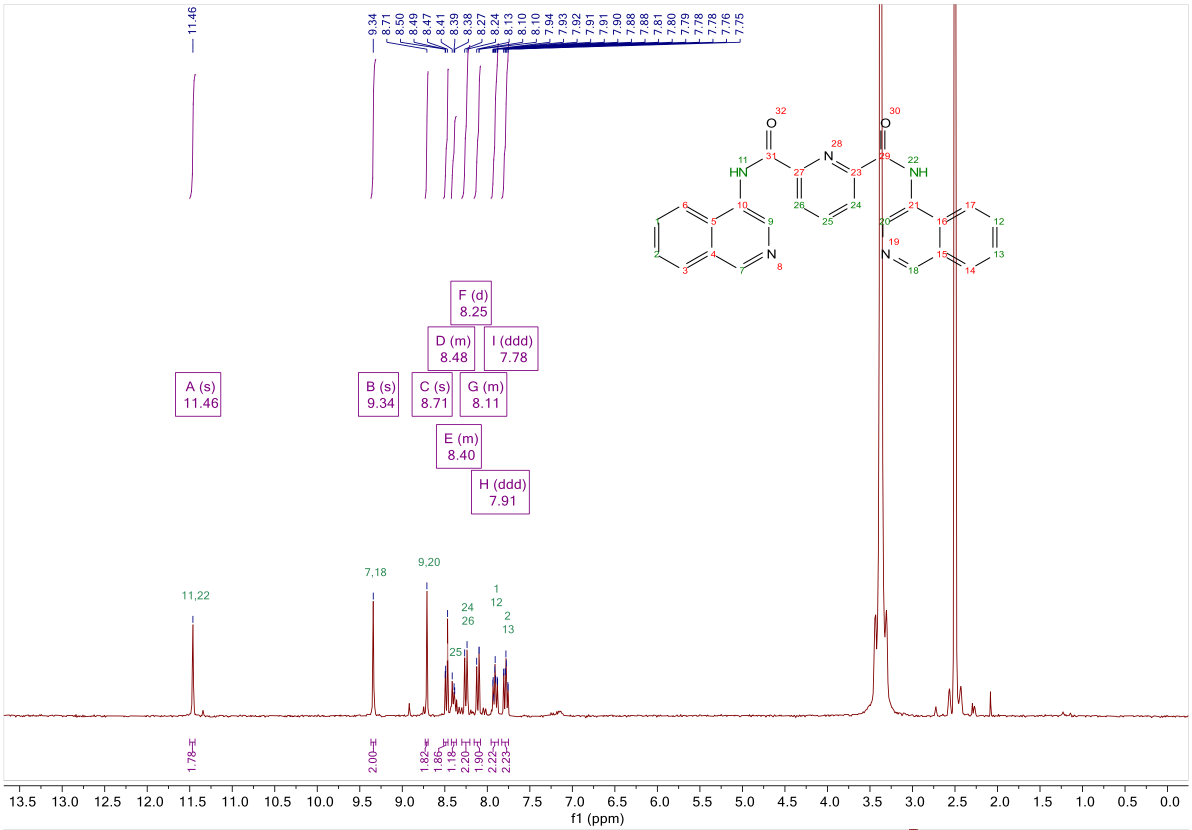

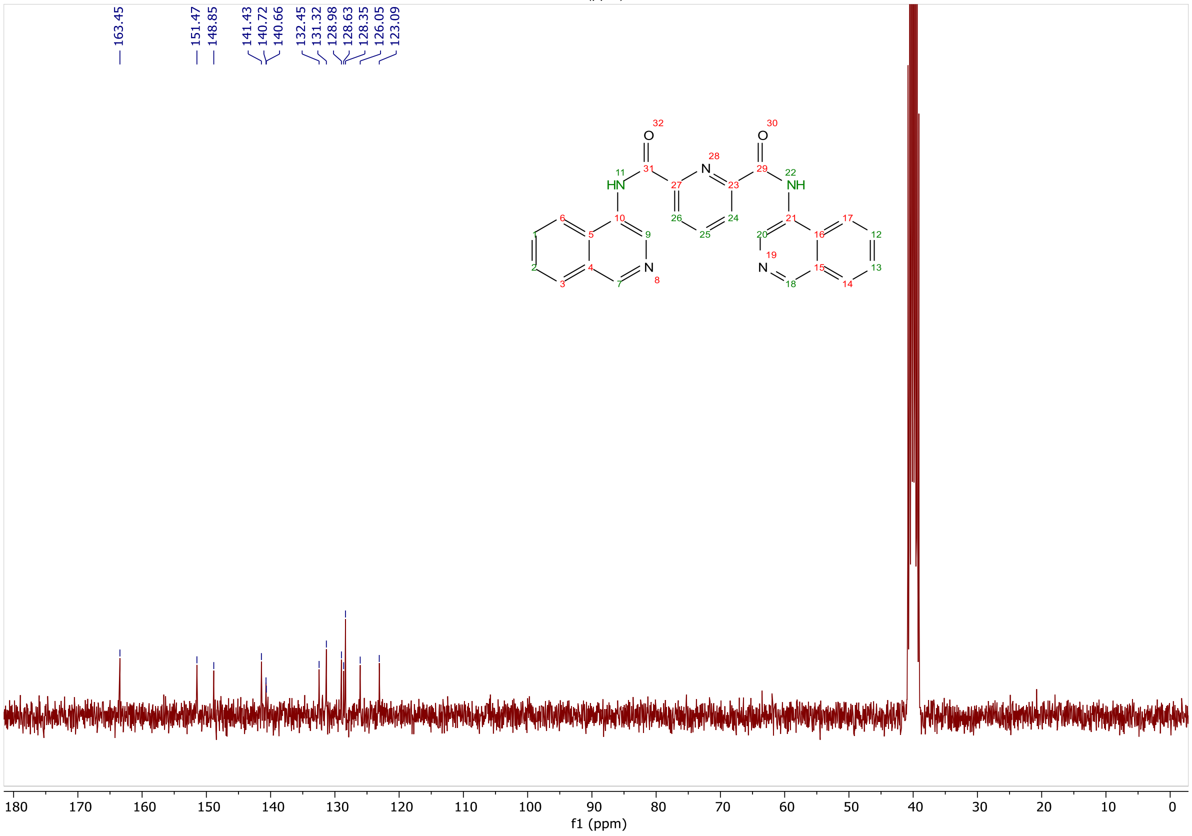

*Figure S20. H-NMR (top) and C-NMR (bottom) spectra of compound **1g**.*

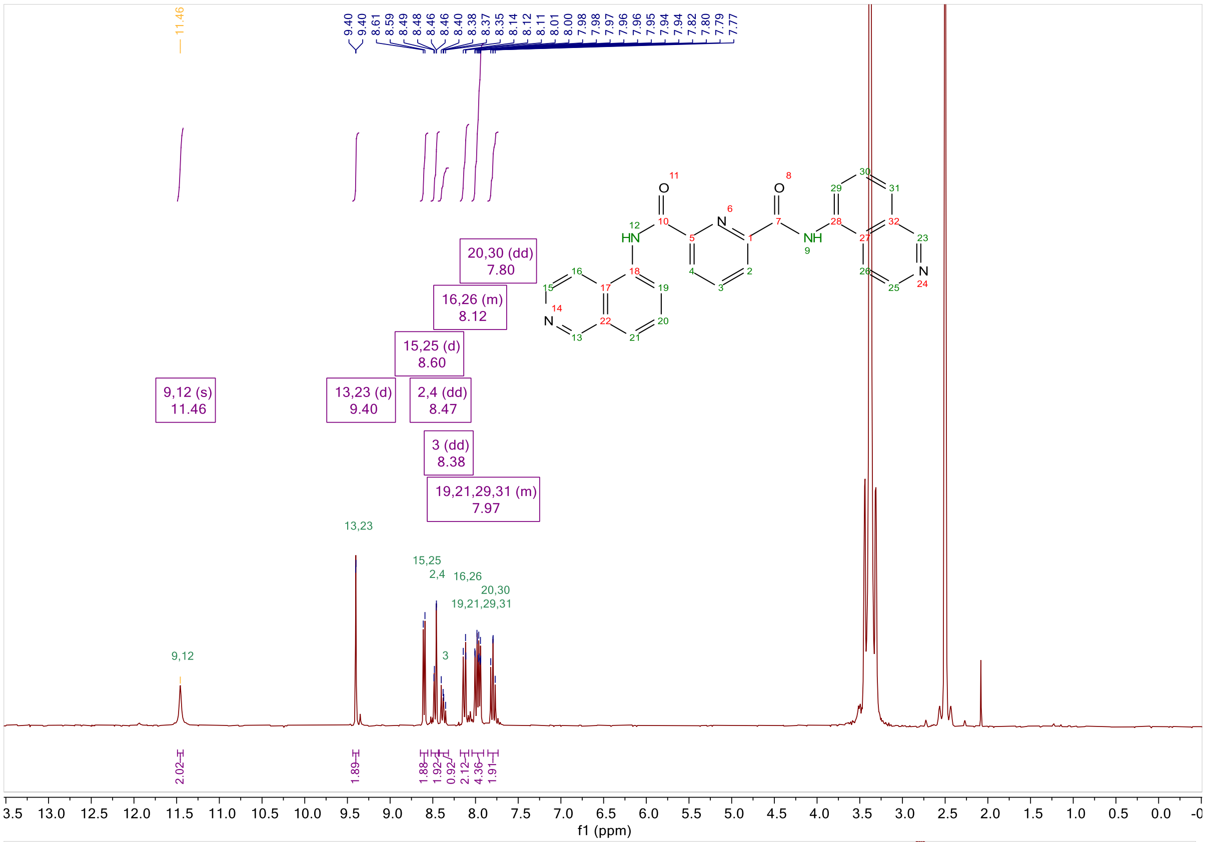


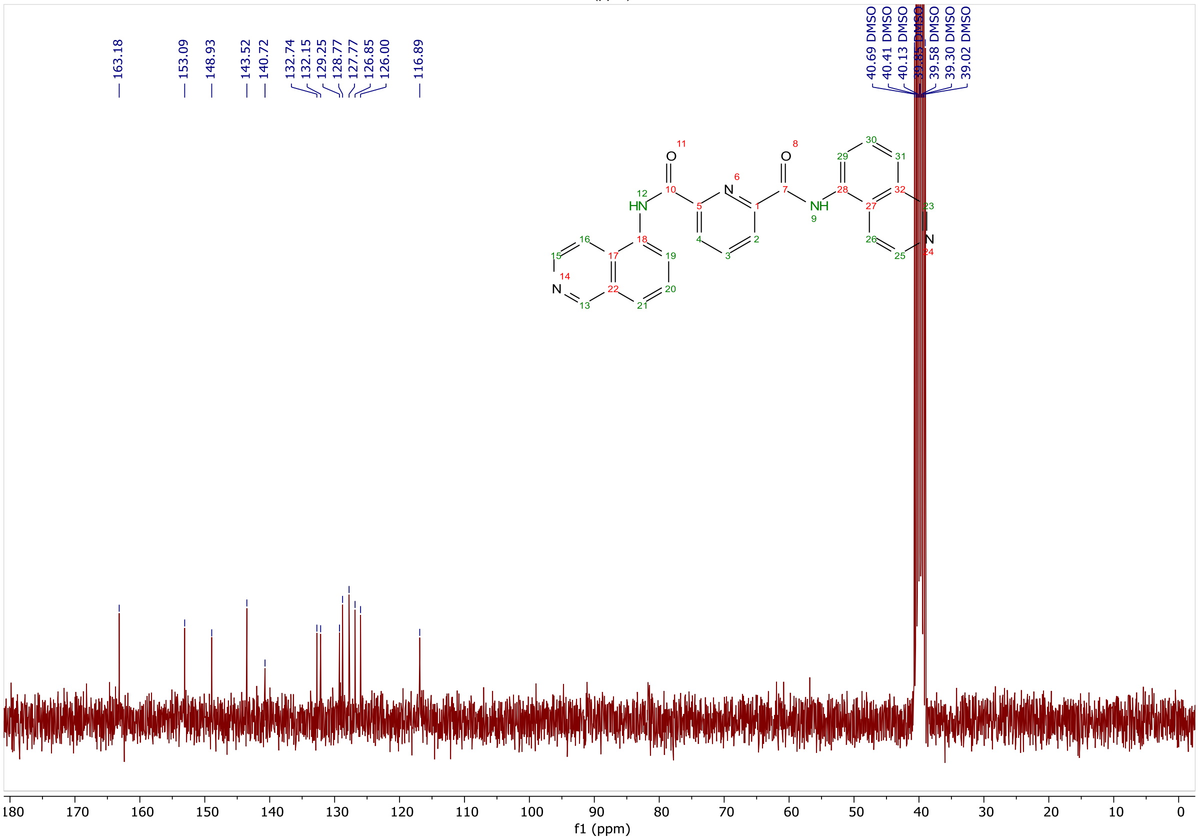


*Figure S21. H-NMR (top) and C-NMR (bottom) spectra of compound **1h**.*

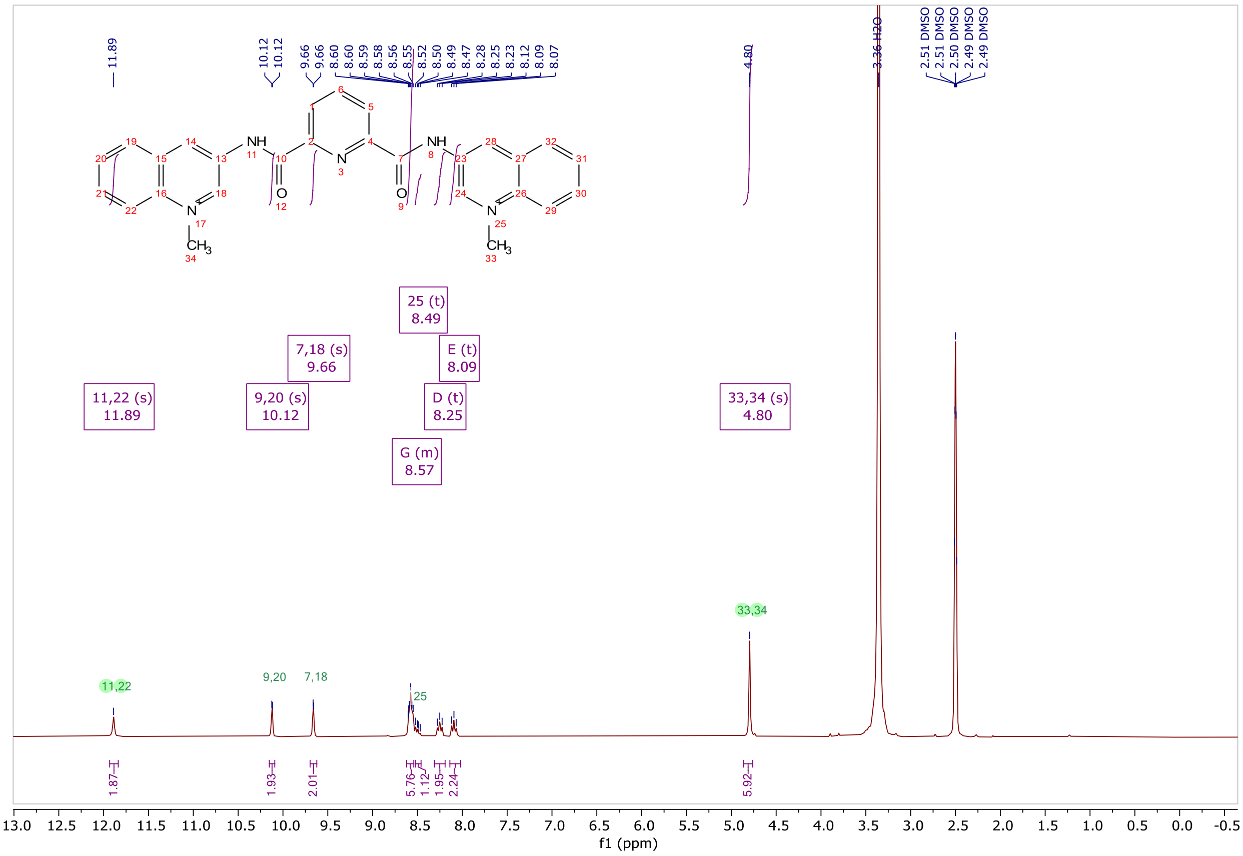


*Figure S22. H-NMR Spectrum of **2a**.*

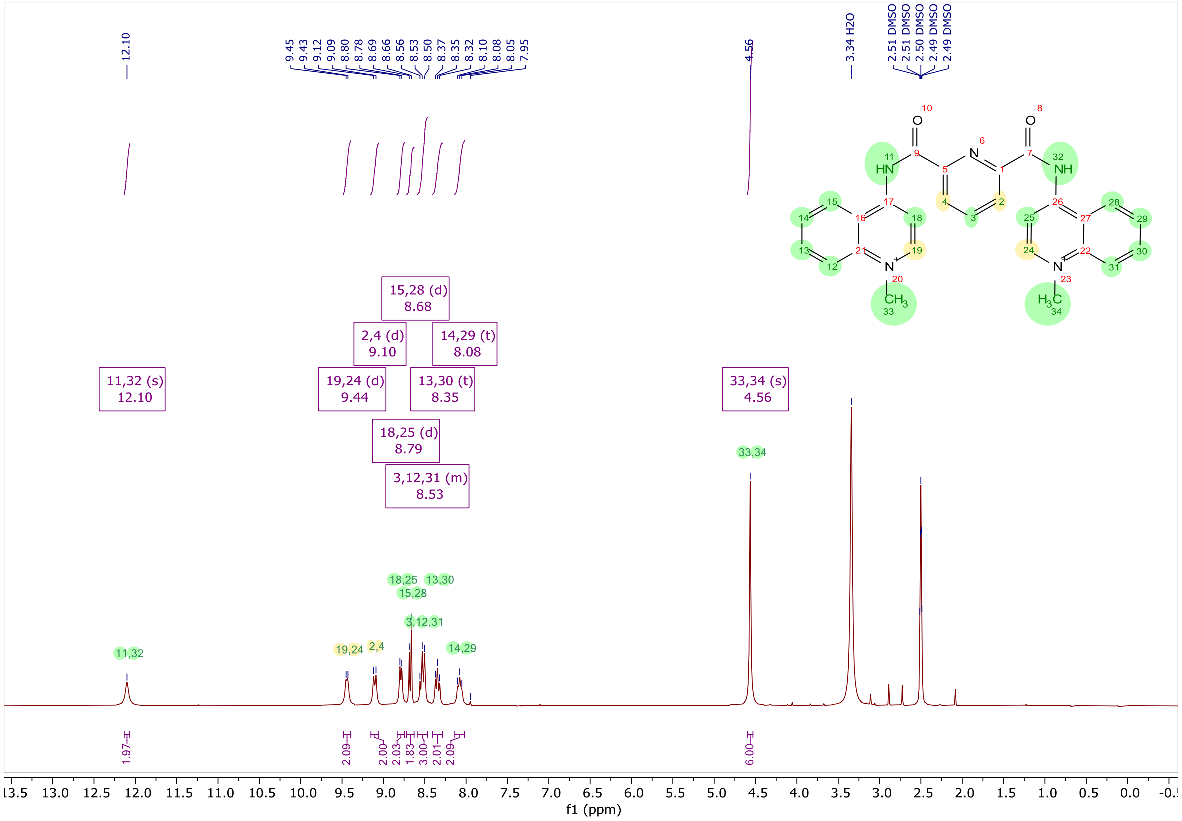


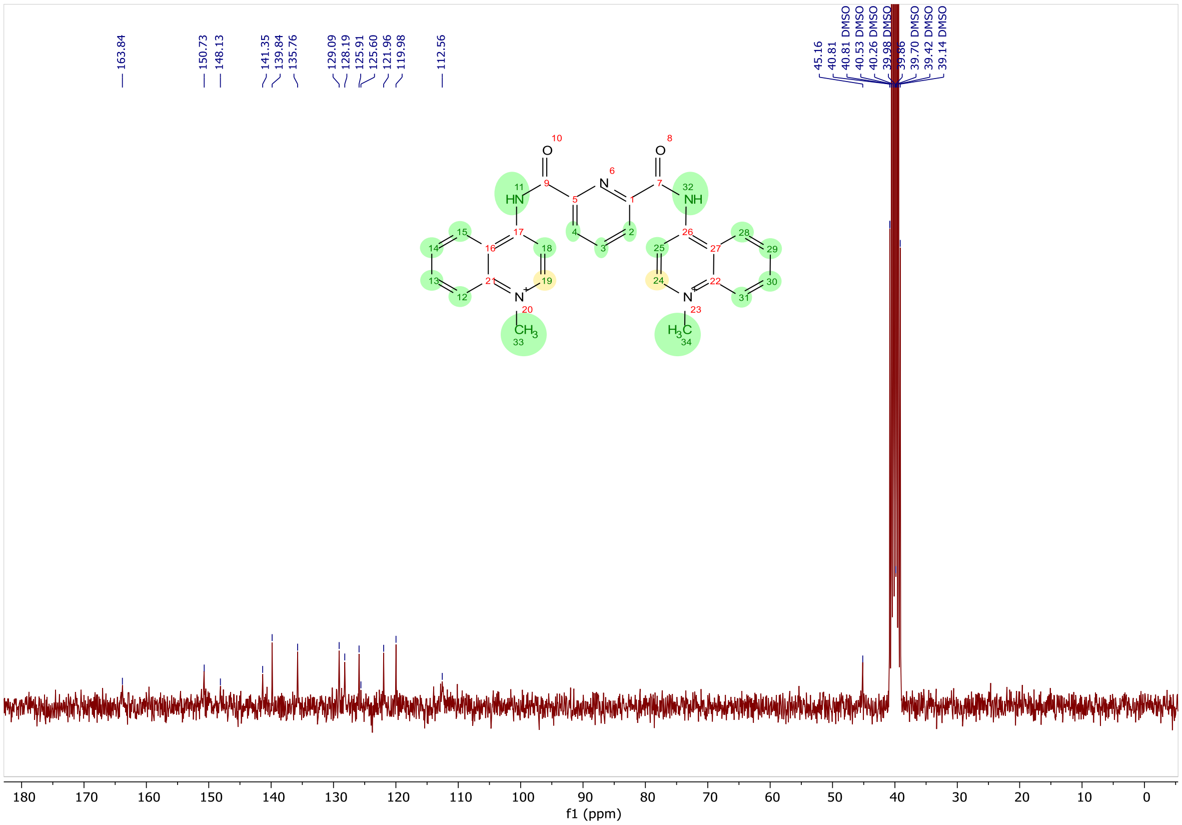


*Figure S23. H-NMR (top) and C-NMR (bottom) spectra of compound **2b**.*

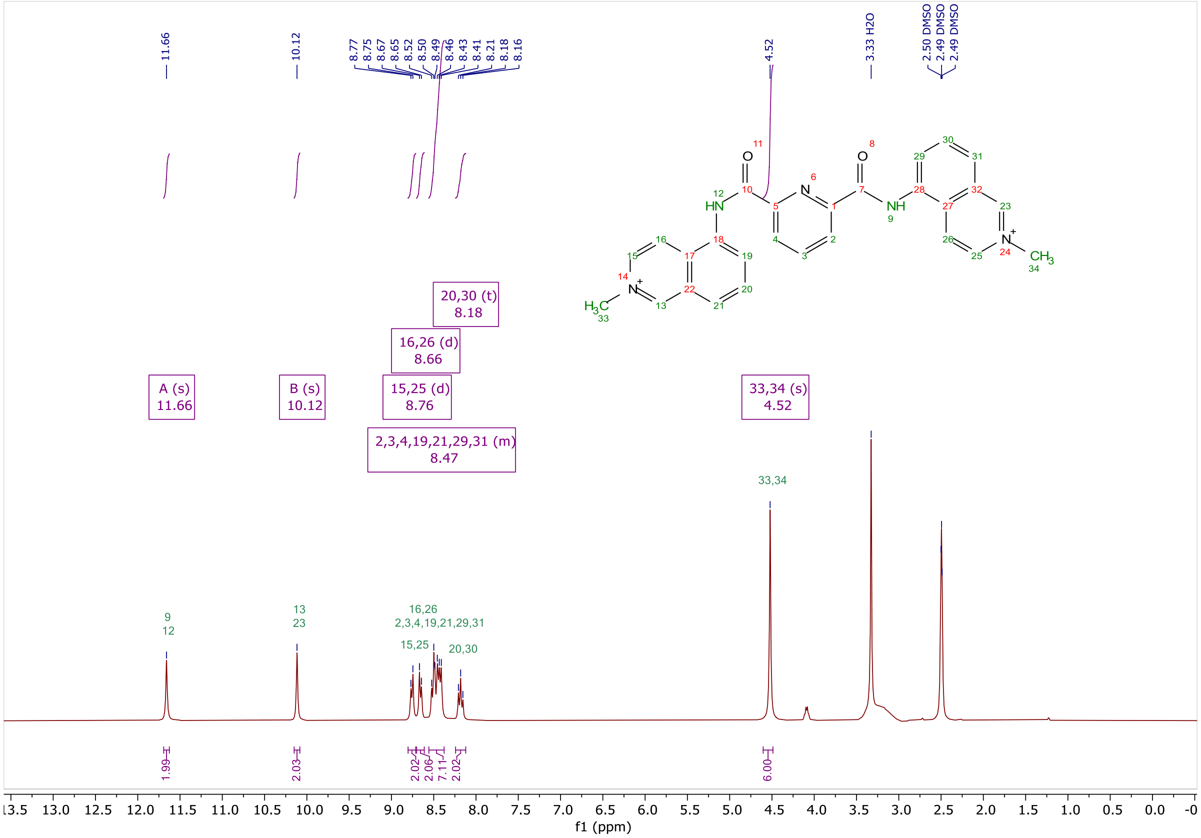


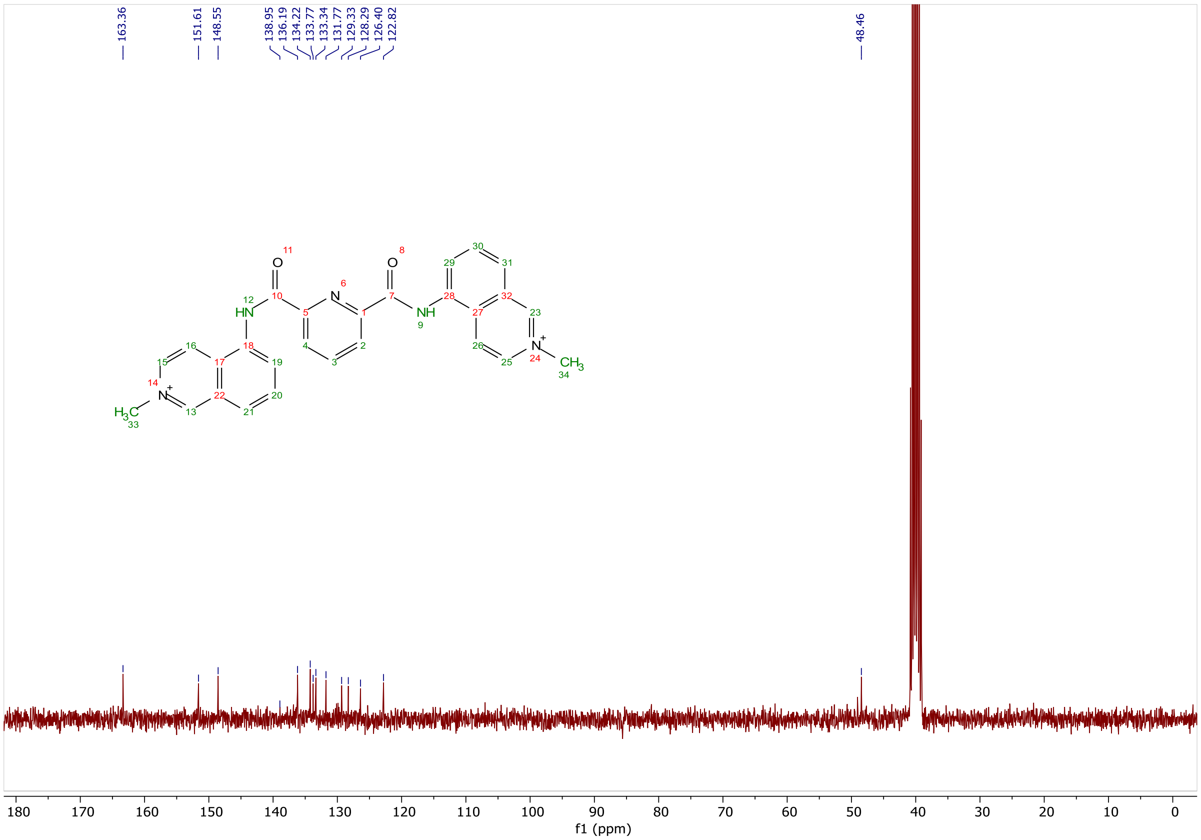


*Figure S24. H-NMR (top) and C-NMR (bottom) spectra of compound **2c.***

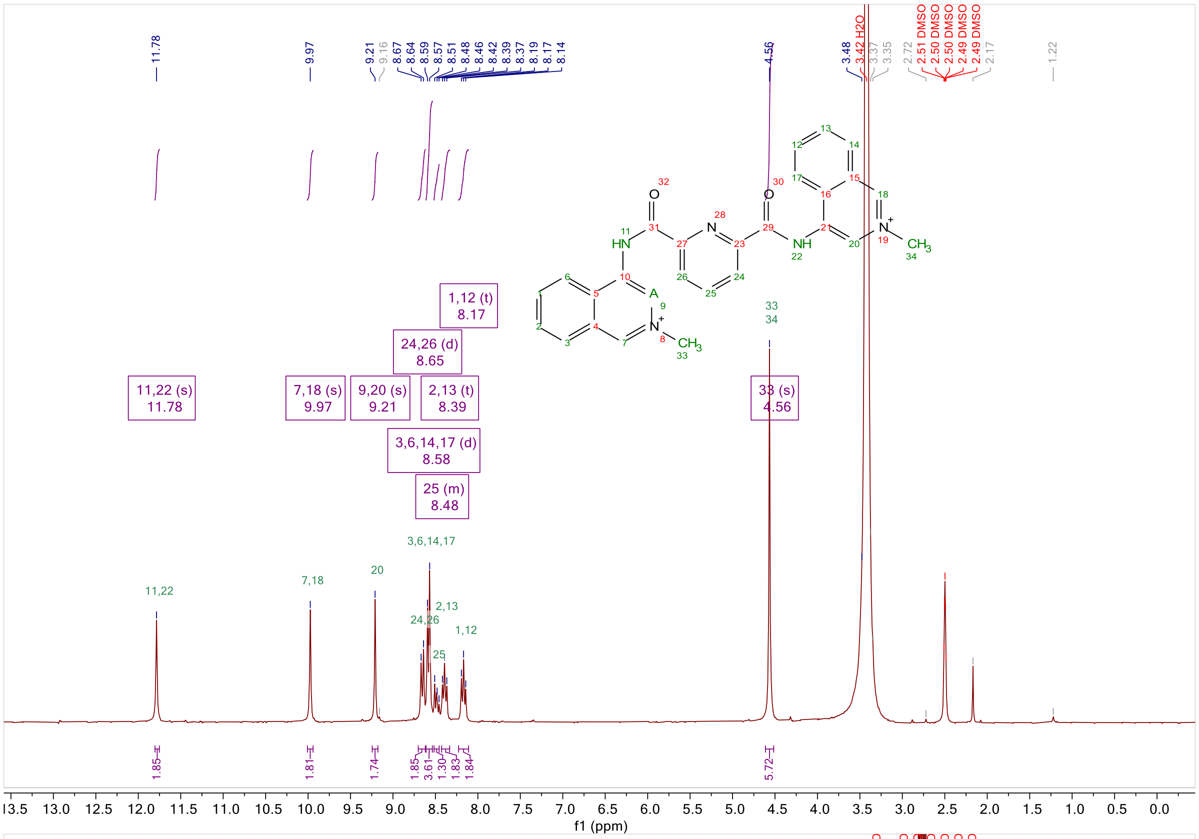


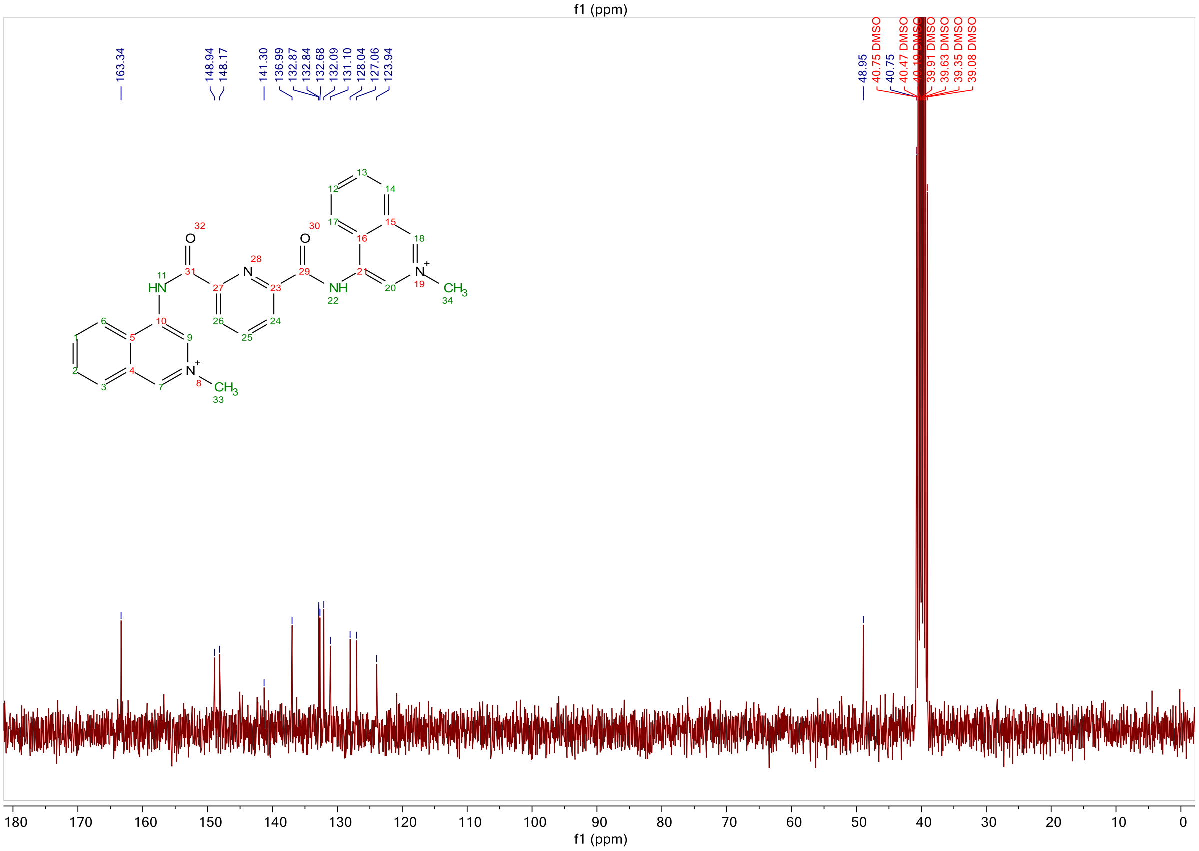


*Figure S25. H-NMR (top) and C-NMR (bottom) spectra of compound **2d.***

# 7. LC-MS Characterization

## Compound **1a**

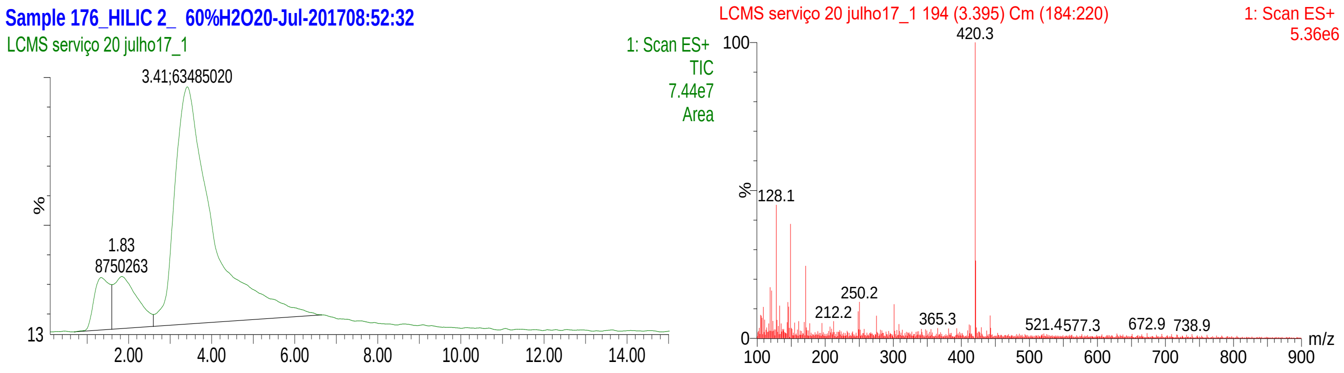


*Figure S26. TIC chromatogram of sample **1a** with peak integration. Mass spectrum of compound at RT=3.395 min.*

## Compound **1b**.

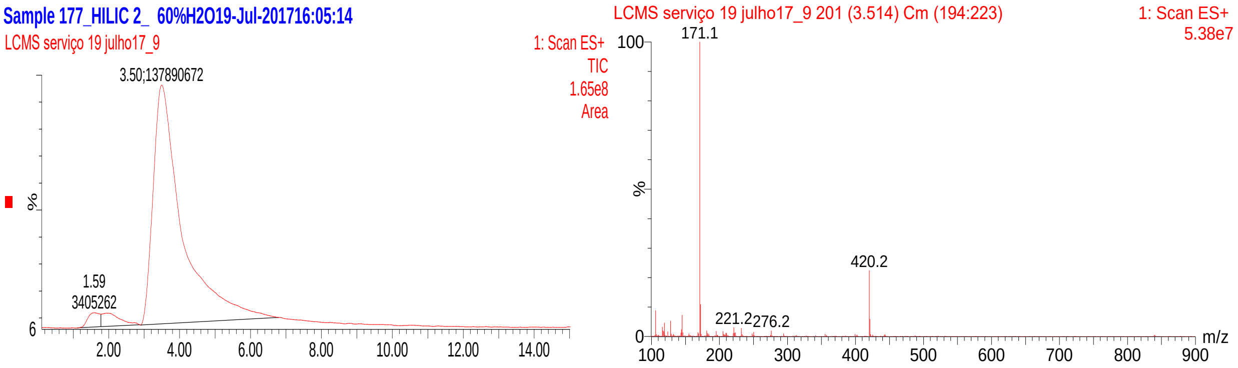


*Figure s27. TIC chromatogram of sample **1b** with peak integration. Mass spectrum of compound at RT=3.514 min.*

## Compound **1c**.

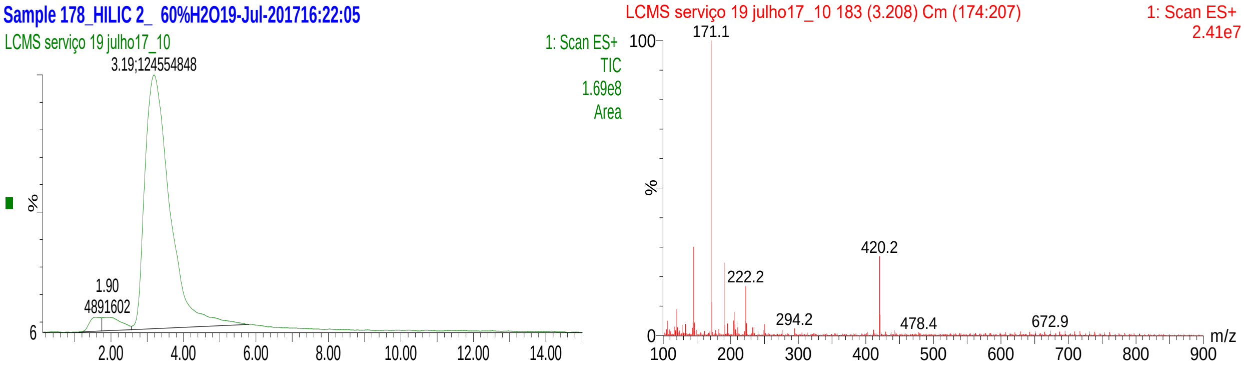


*Figure s28. TIC chromatogram of sample **1c** with peak integration. Mass spectrum of compound at RT=3.208 min.*

Compound **1d**.

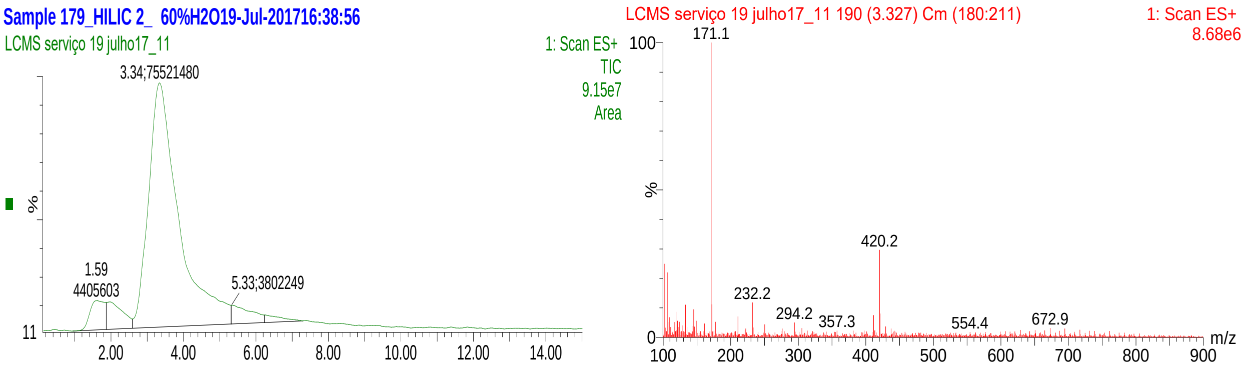


*Figure s29. TIC chromatogram of sample **1d** with peak integration. Mass spectrum of compound at RT=3.327 min.*

Compound **1e**.

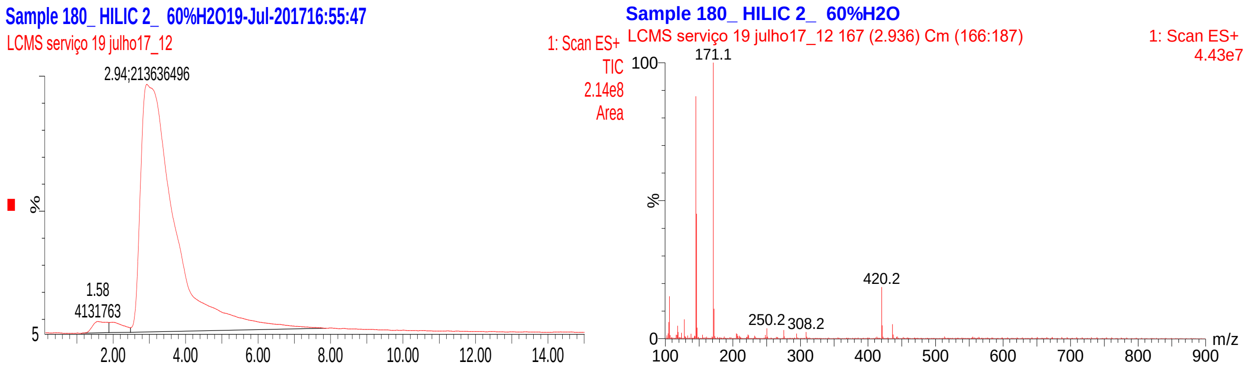


*Figure s30. TIC chromatogram of sample **1e** with peak integration. Mass spectrum of compound at RT=2.936 min.*

Compound **1f**.

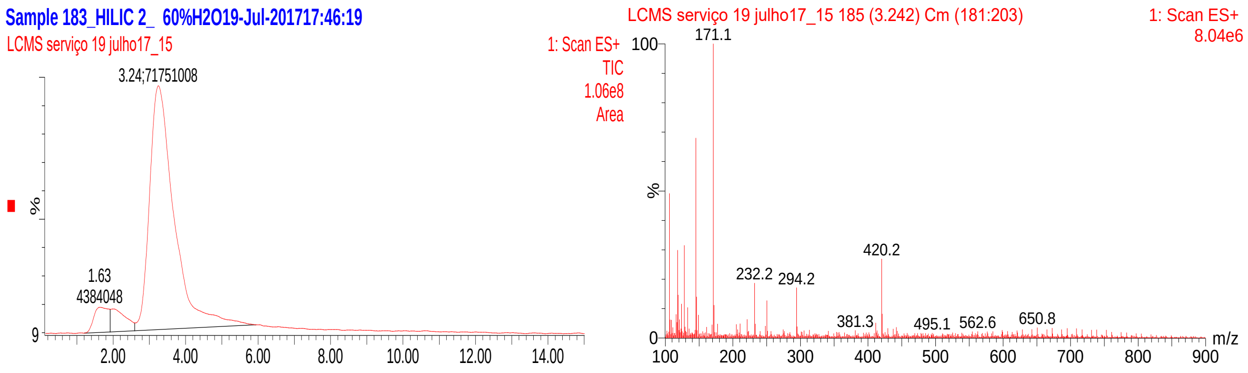


*Figure s31. TIC chromatogram of sample **1f** with peak integration. Mass spectrum of compound at RT=3.242 min.*

Compound **1g**.

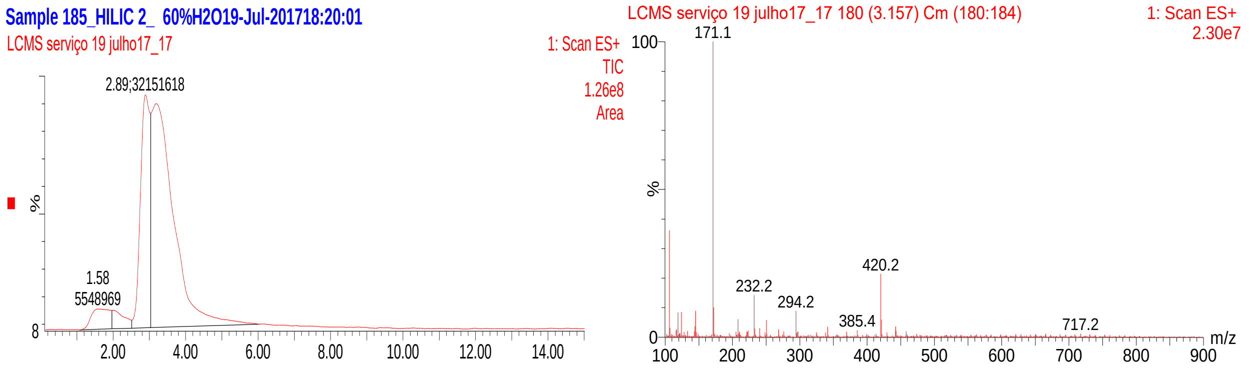


*Figure s32. TIC chromatogram of sample **1g** with peak integration. Mass spectrum of compound at RT=3.157 min.*

Compound **1h**.

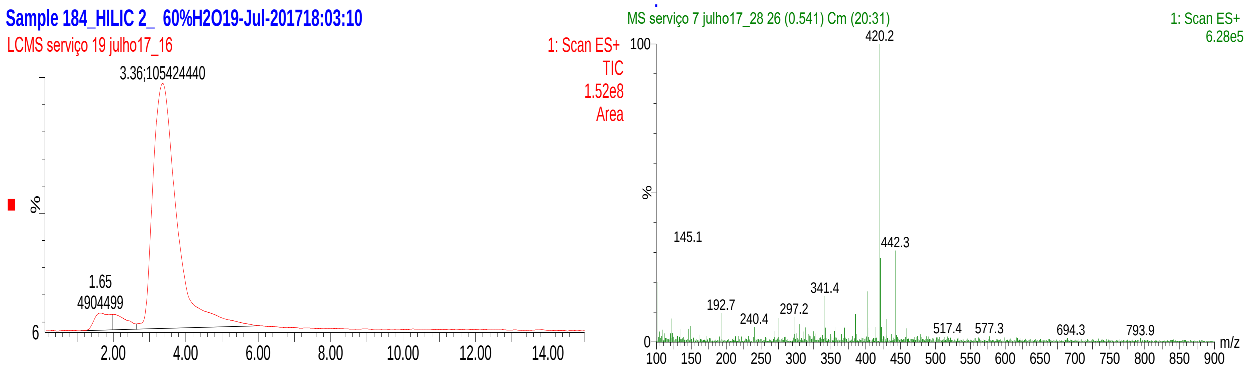


*Figure s33. TIC chromatogram of sample **1h** with peak integration. Mass spectrum of compound at RT=3.365 min.*

## Compound 2a.

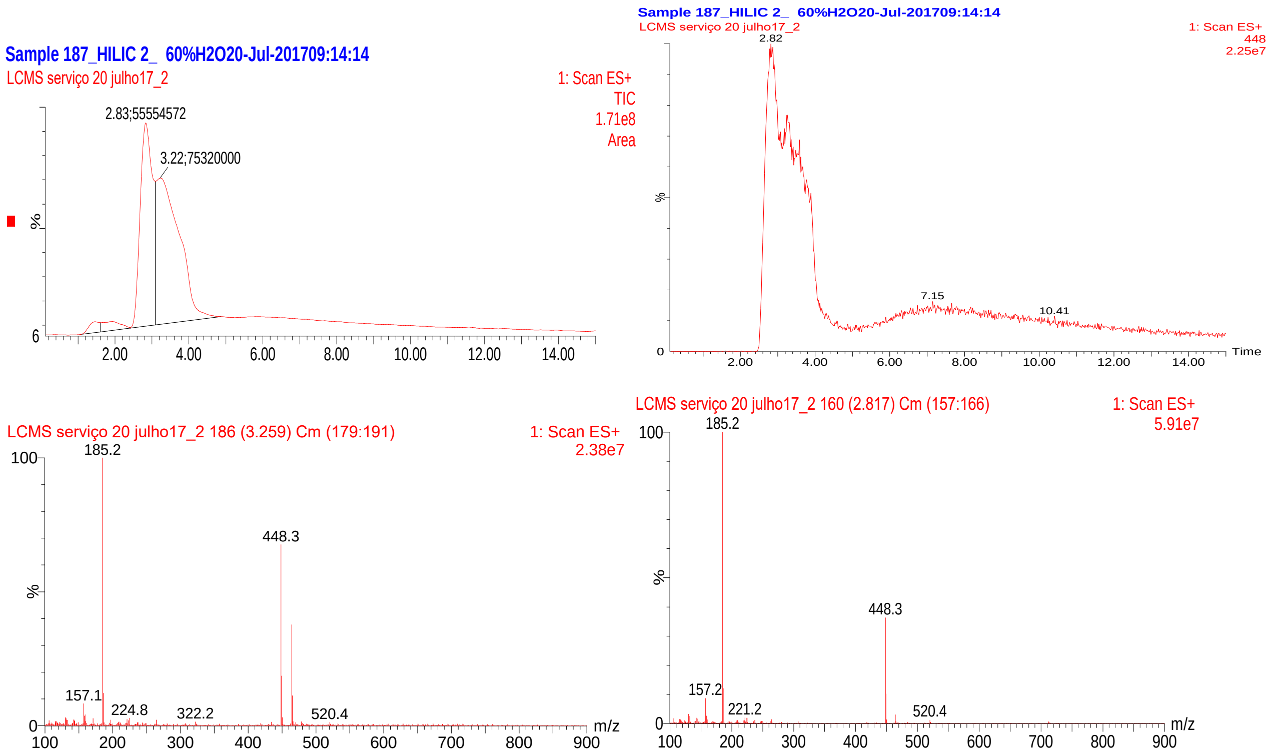


*Figure s34. TIC chromatogram of sample **2a** with peak integration (top left). Extracted chromatogram for ion at m/z 448 (top right). Mass spectra of compounds at RT=3.259 min (bottom left) and RT=2.817 min (bottom right)*

## Compound **2b**.

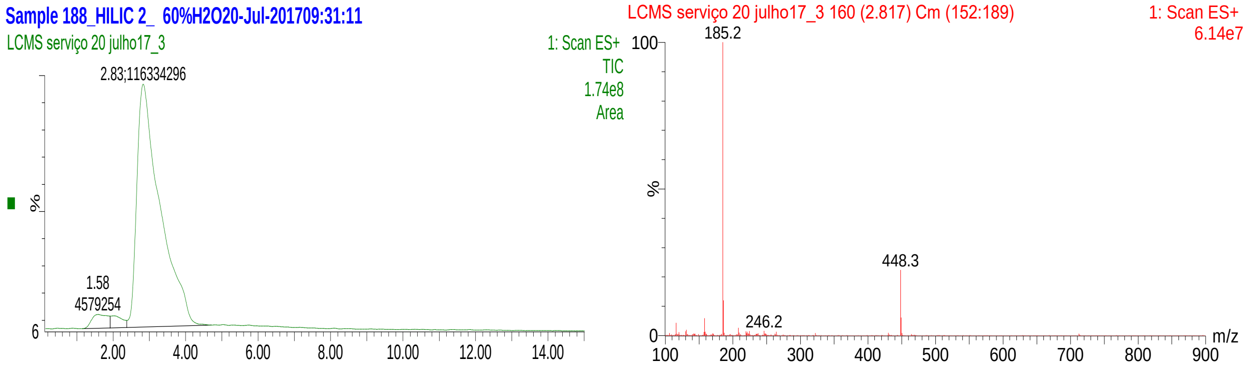


*Figure s35. TIC chromatogram of sample **2b** with peak integration. Mass spectrum of compound at RT=2.817 min.*

Compound **2c**.

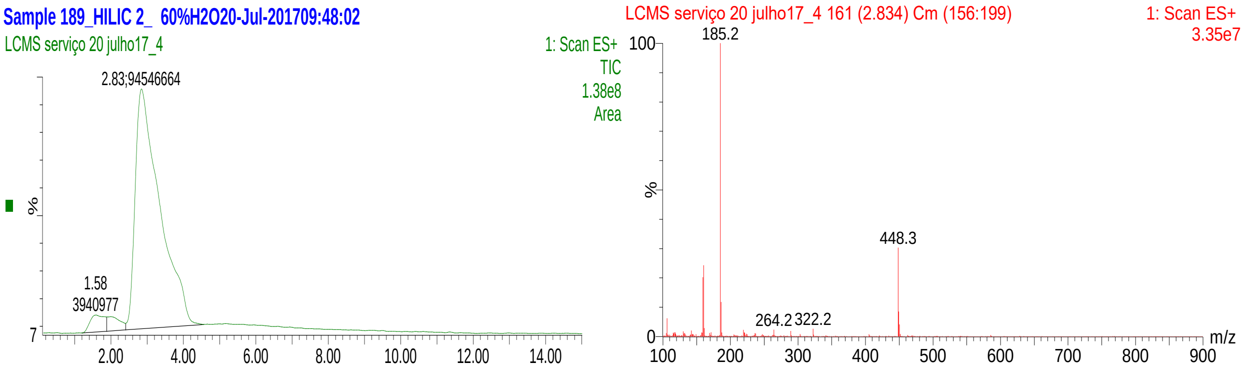


*Figure s36. TIC chromatogram of sample **2c** with peak integration. Mass spectrum of compound at RT=2.834 min.*

Compound **2d**.

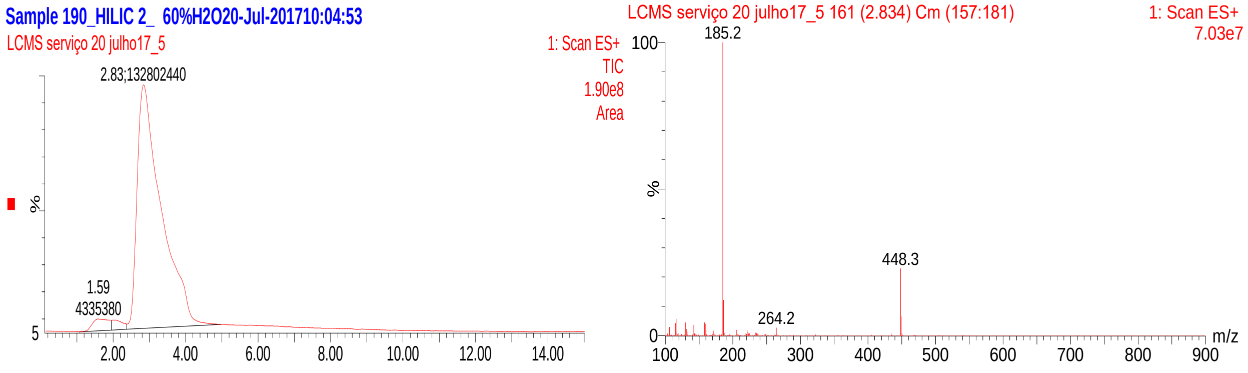


*Figure s37. TIC chromatogram of sample **2d** with peak integration. Mass spectrum of compound at RT=2.834 min.*

## 8. Supplemental references


1. Mendes, E.; Cadoni, E.; Carneiro, F.; Afonso, M.B.; Brito, H.; Lavrado, J.; dos Santos, D.J.V.A.; Vítor, J.B.; Neidle, S.; Rodrigues, C.M.P.; et al. Combining 1,3-ditriazolyl-benzene and quinoline to discover a new G-quadruplex interactive small molecule active against cancer stem-like cells. *ChemMedChem* **2019**, cmdc.201900243, doi:10.1002/cmdc.201900243.

2. Hou, T.; Wang, J.; Li, Y.; Wang, W. Assessing the Performance of the MM/PBSA and MM/GBSA Methods. 1. The Accuracy of Binding Free Energy Calculations Based on Molecular Dynamics Simulations. *J. Chem. Inf. Model.* **2011**, *51*, 69–82, doi:10.1021/ci100275a.

3. Bondi, A. van der Waals Volumes and Radii. *J. Phys. Chem.* **1964**, *68*, 441–451, doi:10.1021/j100785a001.

4. Sitkoff, D.; Sharp, K.A.; Honig, B. Accurate Calculation of Hydration Free Energies Using Macroscopic Solvent Models. *J. Phys. Chem.* **1994**, *98*, 1978–1988, doi:10.1021/j100058a043.
